# Synthesis of Dendritic Oligo‐Glycerol Amphiphiles with Different Hydrophobic Segments to Improve their Performance as Nanocarriers

**DOI:** 10.1002/open.202400448

**Published:** 2025-02-10

**Authors:** Pooja Kumari, Christian Zoister, Natalie Hanheiser, Hesam Makki, Boris Schade, Mathias Dimde, Katharina Achazi, Sumit Kumar, Rainer Haag, Abhishek K. Singh

**Affiliations:** ^1^ Department of Chemistry Deenbandhu Chhoturam University of Science and Technology Murthal 131039 Sonipat India; ^2^ Institut für Chemie und Biochemie Organische Chemie Freie Universität Berlin Takustr. 3 14195 Berlin Germany; ^3^ Institut für Chemie und Biochemie Forschungszentrum fu“r Elektronenmikroskopie Freie Universität Berlin Fabeckstraße 36a 14195 Berlin Germany; ^4^ Department of Chemistry and Materials Innovation Factory University of Liverpool L69 7ZD Liverpool U.K.

**Keywords:** Oligo-glycerol amphiphiles, Hydrophobic, Hydrophilic, Supramolecular assemblies, Encapsulation

## Abstract

A new class of non‐ionic dendritic amphiphiles has been developed from biobased chemicals, in particular glycerol‐based dendrons coupled to commercially available acids *via* the Steglich esterification process. These non‐ionic amphiphiles are functionalized with different hydrophobic segments to investigate the contribution of the same towards their guest transport behaviour. Therefore, different alkyl chains i.e, C8 and C12, as well as two different aromatic units were introduced as a hydrophobic segments and G1‐oligo‐glycerol as a hydrophilic segment. Their physicochemical properties were characterized by different techniques such as dynamic light scattering and fluorescence measurements. The results show that these amphiphiles form a very uniform micellar supramolecular structures that is independent of the hydrophobic system. The critical micelle concentration for the prepared non‐ionic amphiphiles was found to be in the range of 0.3 to 1.8 mg/mL, which depend on the type of hydrophobic units. The encapsulation capacities of the amphiphiles were tested using Nile Red and Nimodipine as model dye and drug, respectively. The encapsulation studies showed a preference for C12‐ and pyrene‐based amphiphiles through relatively different mechanisms unraveled by molecular dynamics (MD) simulations. Further, the cytotoxicity and cellular uptake of these systems as well as the release profiles were investigated.

## Graphical abstract

The synthesized dendritic oligoglycerol amphiphiles with different hydrophobic moieties show efficient micelle formation at low concentrations (CMC: 0.3–1.1 mg/mL) and a strong drug loading capacity, especially for Nile red and nimodipine. Long alkyl chains (C12) improve cargo encapsulation and biocompatibility, while release kinetics suggest potential for controlled drug delivery. These findings serve as a guide for the development of future nanocarriers

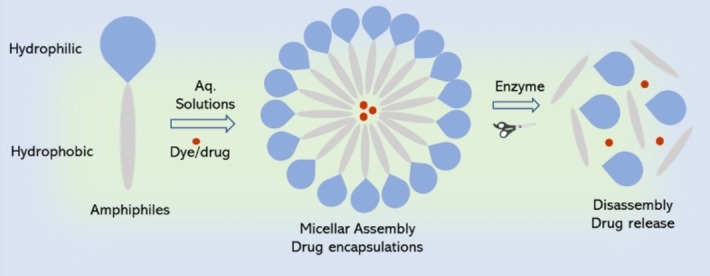

## Introduction

Supramolecular Amphiphiles, a class of molecules possessing the ability to self‐assemble into organized structures in aqueous environments, have garnered enormous interest in recent years due to their wide range of applications in the areas of biomedicine and nanobiotechnology.[^1^, ^2^, ^3^] These supramolecular assemblies, typically consist of hydrophilic and hydrophobic segments, which enables the formation of micelles, vesicles, and other nanoscale architectures through non‐covalent interactions such as hydrophobic effects, ionic interactions, hydrogen bonding, and π–π stacking.[^4^, ^5^, ^6^, ^7^, ^8^, ^9^, ^10^] Designing and development of functional self‐assemblies is among the most important challenges in the realm of supramolecular chemistry. Recently, however, attention has been given to supramolecular carrier systems that carry therapeutic agents in their original, chemically unmodified forms.[^11^, ^12^, ^13^, ^14^] The inherent ability of these supramolecular amphiphiles to self‐assemble and the versatility^[15]^ in structure make them ideal candidates for the creation of nanocarriers that would permit an effective drug encapsulation and delivery to specific target sites in the body. A novel class of amphiphilic systems, dendritic amphiphiles, have attracted substantial attention for their role as functional supramolecular materials.[^16^, ^17^] These dendritic amphiphiles possess well‐defined structures that position them between traditional surfactants and amphiphilic polymers. Their self‐assembly results in well‐defined and stable nanostructures including micelles, vesicles, depending on the specific amphiphile composition.^[1]^ Over the last decade, the design of functional nanocarriers within surfactants and block copolymers has firmly established the structural role of amphiphilic molecules. The structural versatility of amphiphiles arises from their ability to balance hydrophobic and hydrophilic forces, resulting in well‐defined aggregates that can encapsulate and transport a variety of guest molecules. Hence, the structural design of an amphiphilic compound plays a pivotal role in determining their efficacy as drug‐delivery vehicles.[^17^, ^18^, ^19^] In the realm of drug delivery, amphiphilic nanocarriers offer substantial advantages over traditional methods, particularly in addressing challenges related to the low aqueous solubility of drugs, short circulation time and high dosage requirements. In the category of non‐ionic amphiphiles, oligo‐glycerol‐based architectures (G1−G2) have been extensively studied in the literature for their aggregation behaviors and their potential as nanocarriers.[^20^, ^21^, ^22^, ^23^, ^24^] In the current work, the compounds synthesized with G1 dendritic oligo‐glycerol which is functionalized with diverse hydrophobic segments, exhibit distinct structural characteristics that are integral to their function. The oligo‐glycerol core provides a suitable hydrophilicity, thus allowing for solubility and biocompatibility, while variations in the hydrophobic moieties permit modulation of the interactions with the drug molecules to optimize their encapsulation efficiency.^[25]^ We have synthesized four new dendritic oligo‐glycerol non‐ionic amphiphiles, each possessing an identical hydrophilic moiety i. e. G1 oligo‐glycerol but differing in their hydrophobic segments i. e. alkyl chain C8 to C12, and along with aromatic rich Pyrene and Naphthalene unit. These compounds have been successfully synthesized and thoroughly characterized using ^1^H, ^13^C NMR, and Mass spectrometry, confirming their structures and purity. Dynamic Light Scattering (DLS) and Cryogenic Transmission Electron Microscopy (CryoTEM) were used to study the size, distribution, and morphology of the supramolecular assemblies. Additionally, encapsulation and release studies were conducted using Nile Red and Nimodipine as model drugs. The encapsulations study has been supported by molecular dynamics (MD) simulations. Furthermore, the cytotoxicity and cellular uptake properties of the assemblies have been evaluated using A549 cells.

## Results and Discussion

### Synthesis

**Scheme 1:**
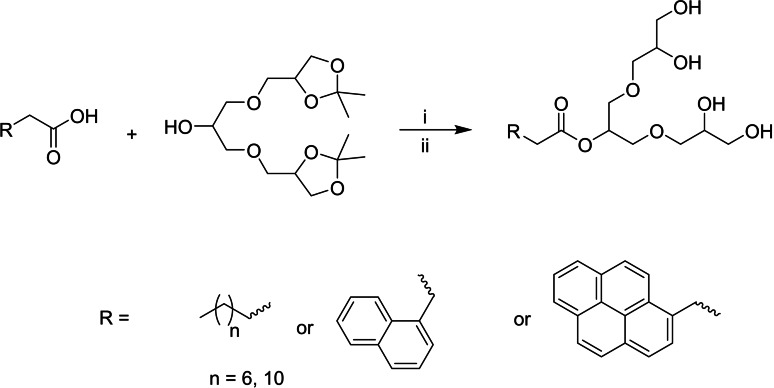
Synthesis of final amphiphiles: i) EDC.HCL, DMAP, DCM: DMF, rt., 24 h; ii) Dowex 50W X8, methanol 50 °C 24 h, C12–G1 (95 %), C8−G1 (85 %), Py−G1 (88 %), Nap–G1 (92 %).

The synthesis of the G1 dendron was initiated using solketal and epichlorohydrin in the presence of NaOH according to a method previously reported in the literature.^[26]^ The synthesis was further advanced using Steglich esterification, in which EDC⋅HCl and DMAP in DCM were used to couple an alcohol and a carboxylic acid via an ester linkage. The crude product was then purified by column chromatography to obtain the acetal‐protected target amphiphiles. The successful formation of the ester bond was confirmed by ^13^C‐NMR, with characteristic peak observed at 173–175 ppm. Subsequently, the acetal‐protected G1 was deprotected with Dowex‐50W X8 in the presence of methanol, resulting in the final dendritic amphiphiles, as shown in Scheme 1. In the ^1^H NMR spectrum, the appearance of the methylene signal at about 5.1–5.2 ppm confirmed the formation of the ester bond in all the four amphiphiles.

**Figure 1:**
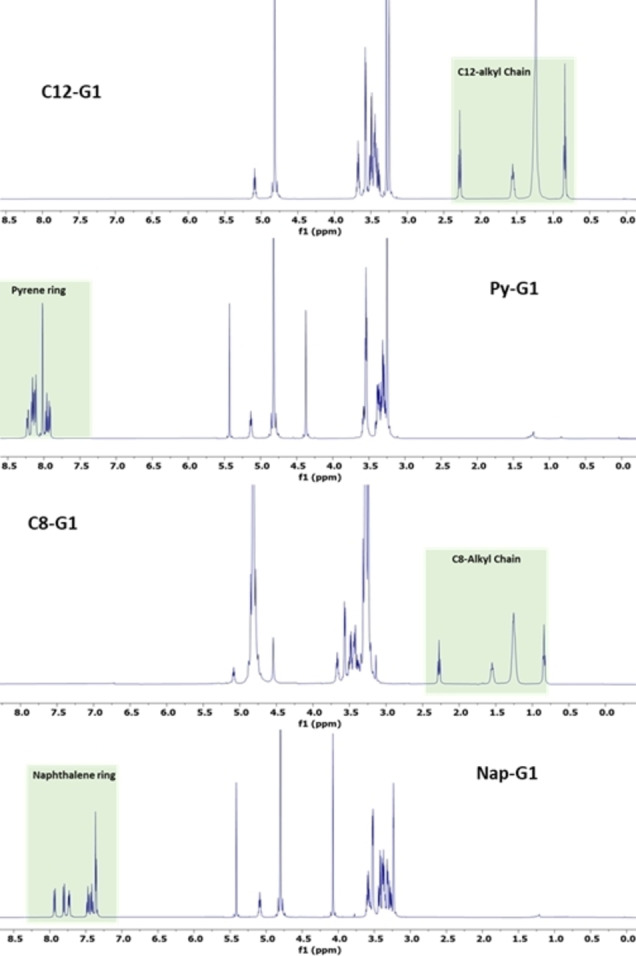
^1^H NMR of the final synthesized amphiphiles. The NMR was performed at 500 MHz.

The C12−G1 and C8−G1 were confirmed by the appearance of peaks in an up‐fielded region between 2.5–0.5 ppm in ^1^H NMR while the peaks in aromatic region 7.5–8.5 ppm confirm the coupling of Pyrene and Naphthalene ring (Figure 1). In addition, the disappearance of the 12H peak in the 1.3–1.5 ppm range indicated successful deprotection of the acetal‐protected G1 dendron (Figure 1). Further, mass spectrometry provided evidence for the formation of the desired products, with the observed mass closely matching the expected molecular weight of the synthesized amphiphiles. Overall, the spectral data confirms the precise structure of the dendritic amphiphiles and underlines the success of the synthesis process (see the figure S1 to S8 in SI).

### Physiochemical Characterization

**Figure 2:**
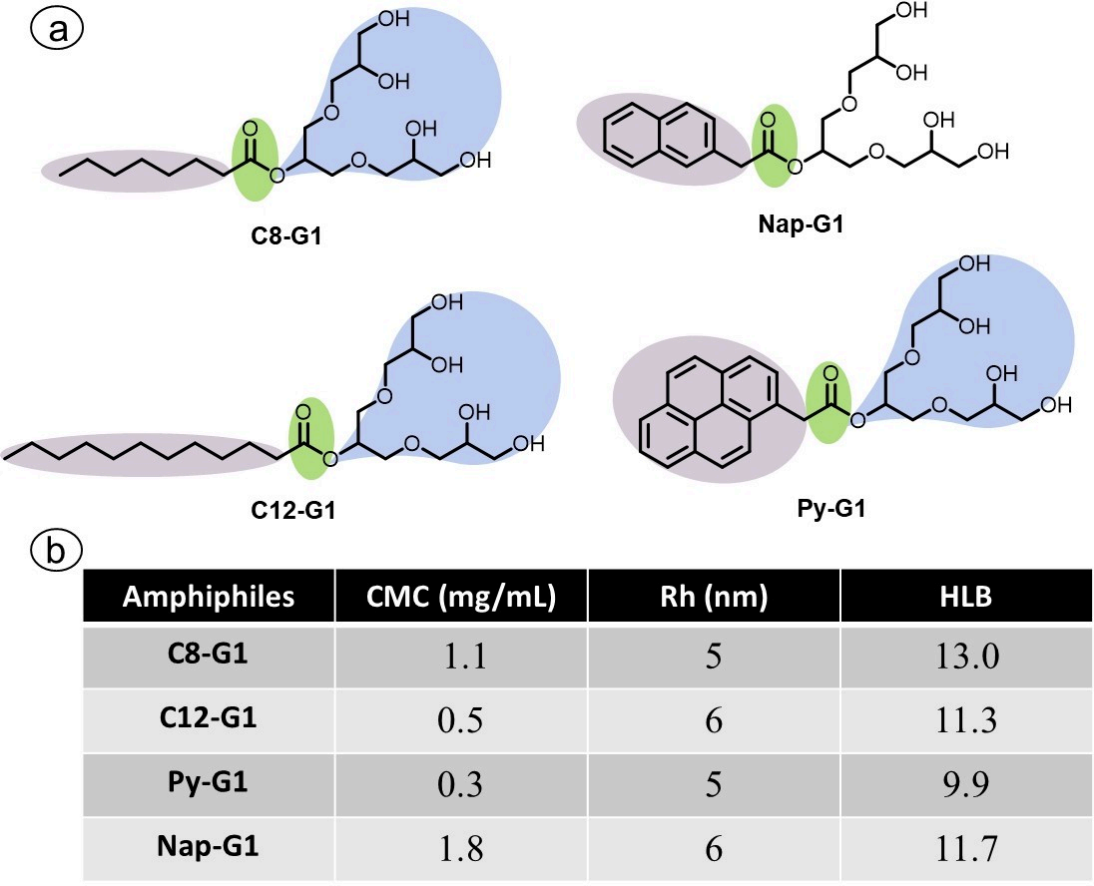
a) Final structure of the synthesized amphiphiles; b) CMC, DLS and HLB.

To understand the physical and chemical properties of the synthesized amphiphiles, their critical micellar concentration (CMC) and aggregation behaviour were evaluated using dynamic light scattering (DLS) and cryo‐transmission electron microscopy (cryo‐TEM). Additionally, the hydrophilic‐lipophilic balance (HLB) values were determined using Griffin's equation^[27]^ to assess solubility behaviour (Figure 2).

### CMC (Critical Micelle Concentration)

The Nile red encapsulation method has been used to determine the CMC of the synthesized molecules.^[28]^ The study of the critical micelle concentration of the synthesized amphiphiles was made by the fluorescence technique using “Nile red” as a model dye. A stock solution of the dye with a concentration of 1 mg/mL was prepared in THF. Further, 10 microliters of stock solution were added in each empty vial followed by complete evaporation of THF to form a thin layer. A stock solution of amphiphiles with the concentration of 5 mg/mL was prepared using Milli‐Q water.

**Figure 3:**
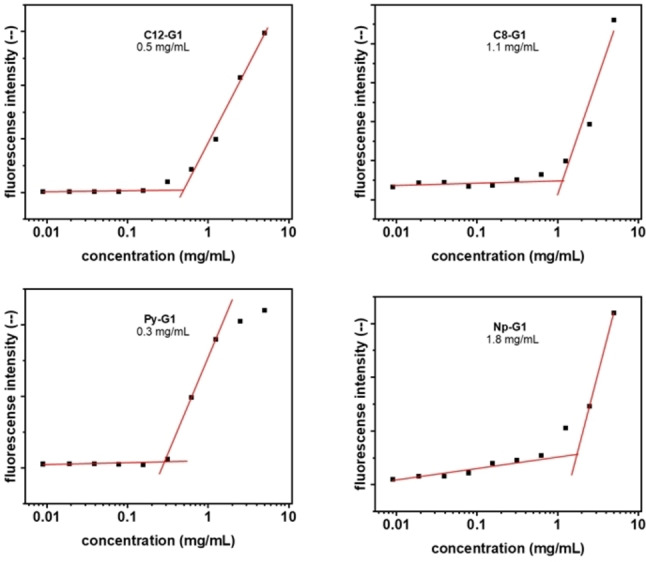
Determination of the critical micellar concentration of amphiphiles using fluorescence spectroscopy using Nile Red as a probe. The Amphiphiles concentration was 5 mg/ml

Twofold serial dilution of the stock solutions was performed to get various concentrations of the amphiphiles, after which it was added to the vials with a thin film of the dye in these vials and overnight stirring. The solutions were subsequently filtered off from the non‐encapsulated dye by filtration through a 0.45 μm polytetrafluoroethylene (PTFE) filter and fluorescence measurements were taken using a fluorescence spectrophotometer. The point of inflection in the plot of fluorescence intensity of encapsulated Nile red vs log [amphiphilic conc] indicates the amphiphile‘s CMC value. For all the synthesized Amphiphiles, the CMC value varied in the range of 0.3 to 1.8 mg/mL depending on the type of hydrophobic segments. The critical micellar concentration (CMC) is significantly influenced by the chemical structure, including the length and nature of the hydrophobic and hydrophilic segments. Among the synthesized amphiphiles, those with C12−G1 and Py−G1 exhibit the lowest CMC values, indicating that they form more stable aggregates compared to the other amphiphiles. The particularly low CMC of Py−G1 than Nap−G1 may be Pyrene aggregates more effectively than naphthalene due to its larger aromatic surface area, which allows for stronger π–π stacking interactions and greater hydrophobic driving forces. (Figure 3).

### DLS (Dynamic Light Scattering) and CryoTEM

**Figure 4:**
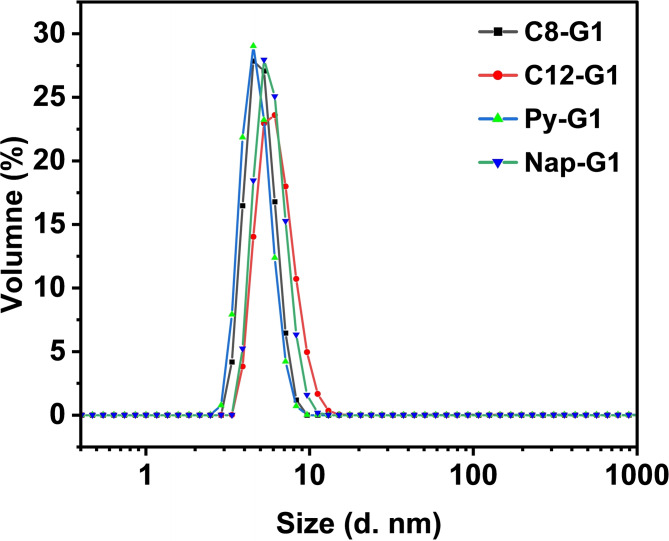
Volume distributions DLS profile of the synthesized amphiphiles at concentration of 5 mg/mL.

Dynamic Light Scattering (DLS) is essential for studying polymers as it offers critical insights into their size distribution and aggregation behaviour in solution. The size distribution of these amphiphilic molecules was evaluated at concentration of 5 mg/mL in aq. solution. The DLS analysis indicated that all the synthesized amphiphiles aggregates in to smaller micelles with range of 5–6 nm (Figure 2&4).

**Figure 5:**
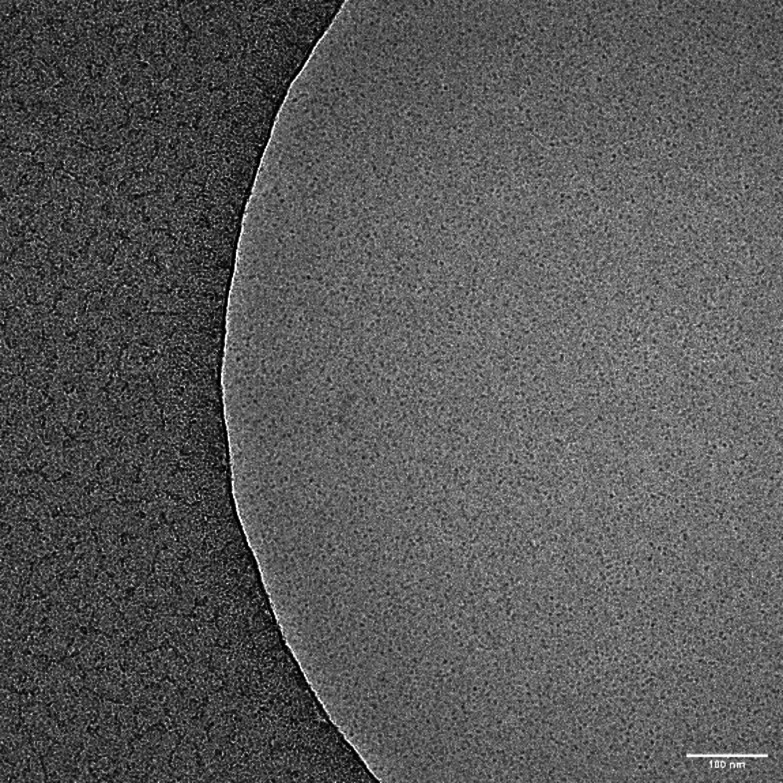
cryo‐TEM micrograph of the C12–G1 amphiphile visualizing small spherical micelles.

The HLB values also reflect to these values which indicate that the these amphiphiles have highly water solubility that result in the lowering the size distributions. These finding was further confirmed by cryo‐TEM in the case of C12−G1, it showed very small micelles that closely match the size distribution obtained from DLS (Figure 5). The Intensity and Number distribution data have been provided in the supporting information as Figure S10.

### Encapsulations

Nile Red and Nimodipine, two hydrophobic guest molecules, were used to test the amphiphiles′ potential as nanocarriers for hydrophobic guests. Nile Red is a fluorescent dye that is environmentally sensitive, and has a low solubility in water.^[29]^ In a lipophilic environment, it displays substantial emission of solvatochromic fluorescence. In nano‐architectures, Nile red encapsulation provides a reasonable amount of information regarding the encapsulation site. On the other hand, A well‐known calcium channel blocker, Nimodipine (NIM) was developed by Bayer AG in 1983 as a derivative of 1,4‐dihydropyridine.

It is applied to both human and animals to enhance cerebral blood flow.[^30^, ^31^] The encapsulation investigations have been carried out using a well‐known thin film technique.^[32]^ The measurements were conducted using an aqueous amphiphilic solution at a concentration of 5 mg/mL. Using a 0.45 μm PTFE filter, the unencapsulated (free) drug/dye that was present in the solution as a precipitate was eliminated.

**Figure 6:**
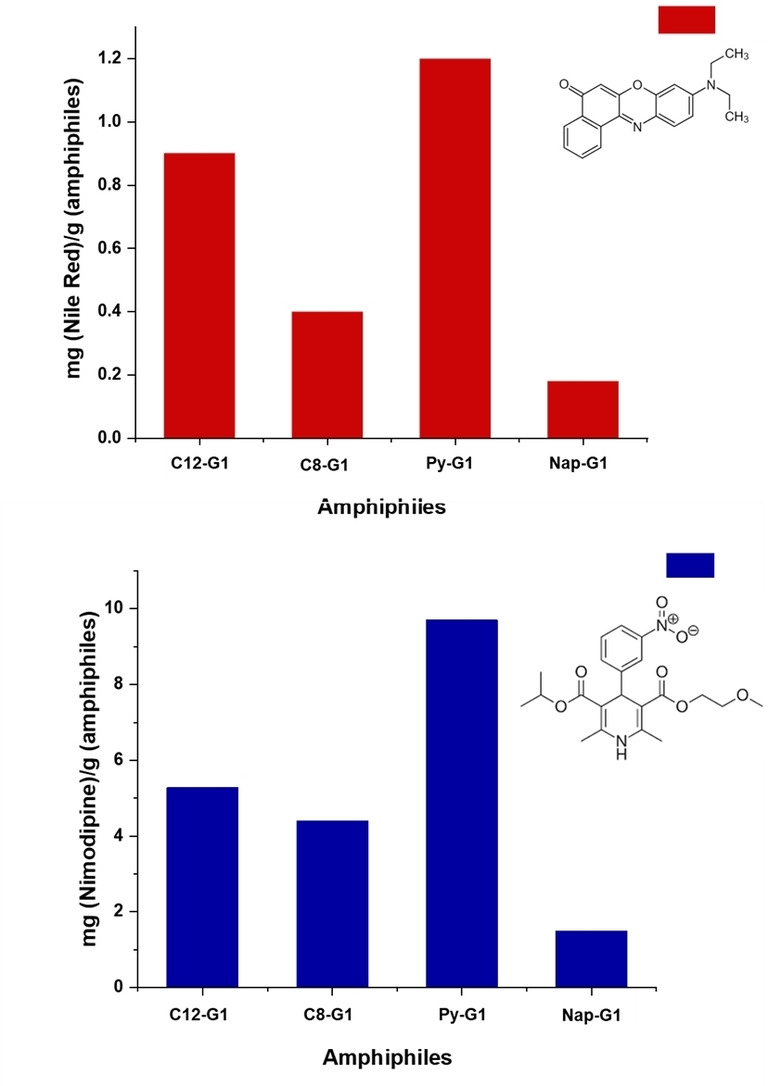
The encapsulation behaviour of synthesized amphiphiles.

The UV absorption spectra (Figure S11 & S12 in SI) confirm the encapsulation of both Nile Red and Nimodipine, with quantification performed using the Lambert‐Beer equation (SI). Figure 6 illustrates the transport capacity of all the amphiphiles, showing a consistent encapsulation pattern for both the drug and dye. C12−G1 and Py−G1 exhibit the highest encapsulation compared to C8−G1 and Nap−G1. The enhanced encapsulation by C12−G1 is likely due to its increased hydrophobicity, while in Py−G1, π–π interactions with both Nile Red and Nimodipine may promote stacking within the nanocarrier cavities. The higher encapsulation efficiency of the C12 alkyl chain compared to C8 and of pyrene compared to naphthalene can be attributed to their structural properties. The longer C12 chain enhances the hydrophobicity, which improves the stabilization and retention of guest molecules in the nanocarrier. Similarly, the larger aromatic surface area of pyrene and the stronger π–π stacking interactions enable better stabilization of hydrophobic drug molecules, resulting in better encapsulation performance compared to naphthalene. These combined factors highlight the importance of both hydrophobic chain length and aromatic ring structure in optimizing the encapsulation efficiency of nanocarriers.

**Figure 7:**
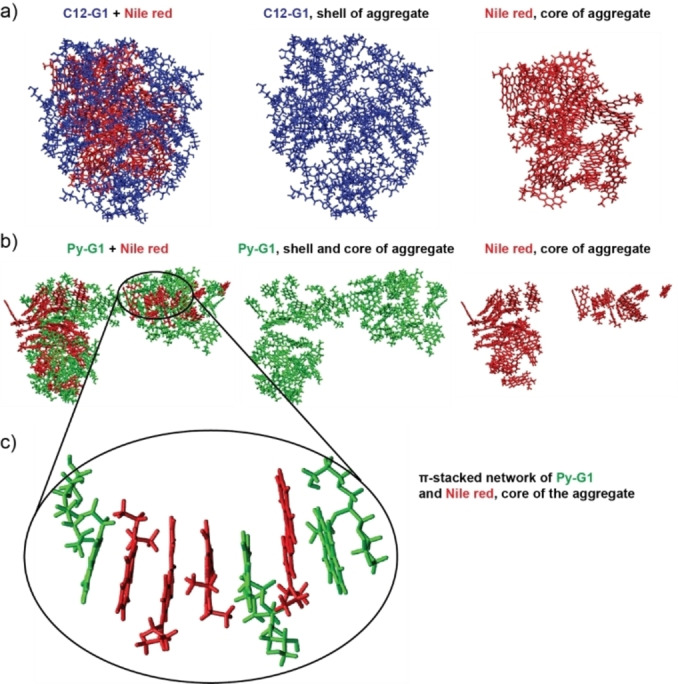
Molecular dynamics (MD) simulations of C12–G1 and Py−G1 with Nile red.

These hypotheses are further supported by molecular dynamics (MD) simulations studies. Atomistic models were developed by simulating the aggregate formation of 50 amphiphile molecules Py−G1 along with 50 Nile red molecules, which were randomly inserted into a simulation box and solvated with water. Figure 7 illustrates the final structure of aggregates. As shown in Figure 7a, C12−G1 forms a shell around a compact π–π stacked aggregate of Nile red. In contrast, Figure 7b, demonstrates that Py−G1 exhibits a strong tendency to form π‐stacking interactions with the drug. While the outer layer of the aggregate is densely populated by Py−G1 molecules, a considerable number of amphiphile molecules are also present in the core of the nanoparticles, creating a π–π stacked co‐continuous network with the drugs (Figure 7c). This behaviour may explain the slightly higher drug encapsulation capacity of Py−G1, as indicated in Figure 6.

### Cytotoxicity Study

**Figure 8:**
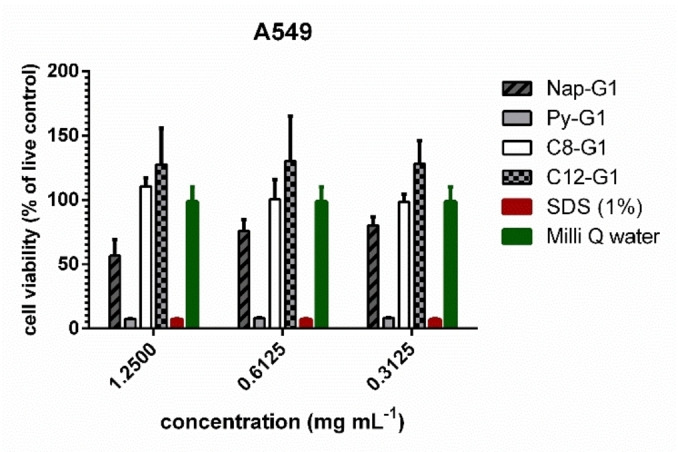
Cell viability profile of amphiphiles on A549 adenocarcinomic (human lung cancer cells) 24 h post treatment using a CCK‐8 assay. Each bar represents the mean value of three technical repeats with SD.

To analyse the cytotoxicity of the Amphiphiles, they were subjected for cell viability evaluation using A549 cell lines. All the amphiphiles show no toxicity at 1.25 mg/mL concentration except Py−G1. (Figure 8). Although the compound with C8 and C12 showed better biocompatibility than the aromatic moieties. The compound was further tested at higher concentrations and the results are shown in SI as Figure S9.

### Cellular Uptake Study

**Figure 9:**
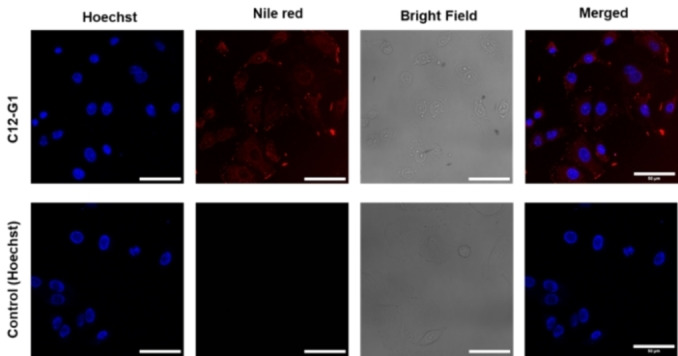
Confocal laser scanning microscopy images of A549 cells after 4 h incubation with Nile Red encapsulating amphiphile (C12–G1). In the images, Nile Red is shown in red and nucleus stained with Hoechst 33342 is shown in blue. The bright field channel is shown in gray scale. The scale bar corresponds to 50.0 μm.

The cellular uptake of Nile red encapsulating amphiphiles was performed using A549 human lung cancer cells and investigated by confocal laser scanning microscopy (cLSM). Considering the cytotoxicity results, amphiphile C12−G1 at a final test concentration of 0.5 mg/mL was chosen for the further cellular uptake studies. Cellular uptake in the cytosol of the cell was seen after first 4 hours of incubation (Figure 9). These results indicate that the amphiphile is well suited for carrying drugs in the cytosol of the cell.

### Release Study

**Figure 10:**
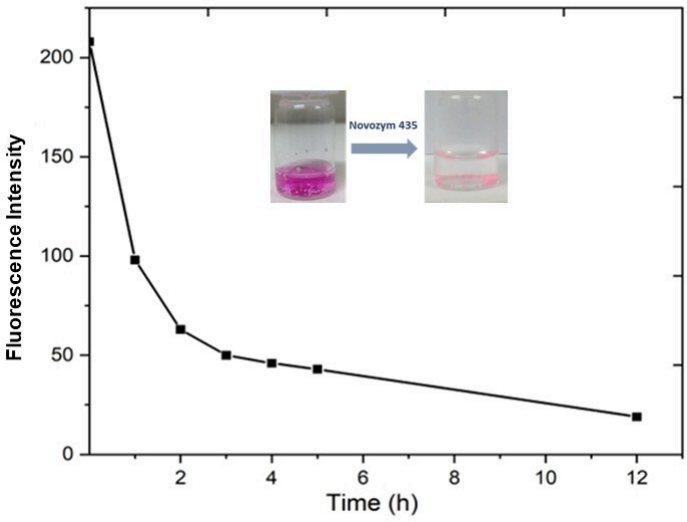
Release profile of the Nile red encapsulated C12–G1 amphiphiles. The experiment was performed at 37 °C at 7.4 pH.

In order to liberate the encapsulated Nile red, the enzymatic cleavage of the ester bond present in the synthesized dendritic amphiphiles was studied. Candida antarctica lipase B was used in a general ester‐hydrolysis procedure to carry out enzyme‐mediated cleavage. Compound C12/G1 has been used to examine Nile red‘s releasing characteristics. First, Nile red was encapsulated in C12−G1 Amphiphile using the same procedure as described in the dye/drug encapsulation section. Further, by following the literature^[33]^ Novozyme 435 and a 20 ul of n‐butanol was added to encapsulated sample and stirred for at 37 °C. At regular intervals, the fluorescence intensity was measured to monitor the nanocarrier‘s release behaviour. It has been observed that most of the Nile red has been released from the micelle in 12 h (Figure 10).

## Conclusions

The synthesized dendritic oligo‐glycerol amphiphiles, featuring a hydrophilic core and varied hydrophobic moieties, were easily synthesized and purified, then thoroughly validated using various characterization techniques. The critical micelle concentration (CMC) values, ranging from 0.3 to 1.8 mg/mL, demonstrate the efficiency of these amphiphiles in forming micellar structures at low concentrations, highlighting the crucial role of the hydrophobic core in supramolecular assembly and guest encapsulation behaviour. The results also indicate the importance of long alkyl chains (C12) and π–π stacking in enhancing cargo encapsulation. Cell viability data revealed that Py−G1 at higher concentrations are toxic to cells, suggesting that long alkyl chains may be preferable over aromatic units for biocompatibility.

Our encapsulation tests with Nile Red and Nimodipine confirmed the strong drug‐loading capacity of the amphiphiles, with complete dye release observed after 12 h. This suggests that the release kinetics can be tuned for time‐dependent release, making these amphiphiles promising candidates for controlled drug delivery systems. Additionally, the nature of the hydrophobic chains significantly impacted micelle formation and stability, underscoring the importance of varying the hydrophobic segments to improve nanocarrier performance. These findings pave the way for future designs, from chemical synthesis to supramolecular behaviour, in creating more effective nanocarriers.

## Experimental Section

### Materials

All the chemicals and solvents used were procured from Sigma‐Aldrich Chemicals, USA. Immobilized Candida antarctica lipase (Novozym 435) was obtained from Novo Nordisk A/S Denmark. For the encapsulation studies the dyes/drugs used were bought from Sigma‐Aldrich Chemicals, USA with determined purity. To monitor the reaction progress, Pre‐coated TLC plate (Merck silica gel 60F254) was used and KMnO_4_ staining solution was used to visualize the spots on TLC plate. Silica gel (60–120 mesh) was used for column chromatography. Millipore water was used for preparation of samples for their physico‐chemical characterization and transport studies. For the cell toxicity, 96‐well flat plate transparent (Sarstedt 96 Flat Transparent Cat. No.: 83.3924), DMEM (1X)+GlutaMAX^TM^ (Gibco, Ref. No.: 31966‐021)+10 % fetal bovine serum (FBS) (Sigma‐Aldrich, Co., Cat. No.: F7524)+1 % penicillin‐streptomycin (PS) (Sigma‐Aldrich, Co., Cat. No.: P4333), Phosphate buffer solution (DPBS, w/o Calcium, w/o Magnesium) (pH=7.2) (PAN‐Biotech, Cat. No.: P04‐36500), Cell lines: both cell lines were obtained from Leibniz Institute DSMZ‐ German Collection of Microorganisms and Cell Cultures GmbH, A‐549 ACC 107, CCK‐8 Kit (Hycultec, Art. No.: HY‐K0301), Tecan SPARK Plate Reader (Tecan Austria GmbH, Ref. No.: 30086376) have been used. And for cellular uptake μ‐slides 8 Well ibidi, 1x μ‐Slide 8 Well ibiTreat (Cat.No: 80806), Hoechst 33342 (Life Technologies GmbH, Darmstadt, Germany), c=10 mg/mL stock solution in DMSO (stored at −20 °C, protected from light), Nile Red, c=0.002 mg/mL stock solution in DMSO (stored at 4 °C, protected from light), Confocal laser scanning microscope Leica DMI6000CSB SP8 (Leica, Wetzlar, Germany) have been used.

### Critical Aggregation Concentration (CAC) Measurements

Synthesized amphiphiles’ critical aggregation concentration was studied by fluorescence measurement technique using ‘Nile red’ as a model dye. A stock solution of the dye was prepared in THF at the concentration 1 mg mL^−1^. To form thin layer, 10 μL of the stock solution was added to each empty vial followed by complete evaporation of THF. The stock solutions of amphiphiles (1 mM) were prepared in Milli‐Q water. To achieve different concentrations of the amphiphiles, two‐fold serial dilution of the stock solutions was done and the solution was then transferred to the vial having thin film of the dye and stirred overnight. Polytetrafluoroethylene (PTFE) filter (0.45 μm) was used to remove the non‐encapsulated dye from the solutions with subsequent fluorescence measurements using Cary Eclipse fluorescence spectrophotometer. The plot of fluorescence intensity maxima values against log [amphiphile concentration] for different samples was used for calculating the CAC value.

### Dynamic Light Scattering (DLS)

Malvern Zeta sizer Nano ZS analyzer integrated with 4 mW He−Ne laser, λ=633 nm, using backscattering detection (scattering angle θ=173°) with an avalanche photodiode detector, was used for determining the size of nanostructures (micelles/aggregates) formed by the supramolecular organization of amphiphiles in the aqueous solution (Milli‐Q water) at a concentration of 5 mg mL^−1^. The samples were then further allowed to mix at 25 °C for 20 h with vigorous stirring. The obtained solutions were then filtered through 0.45 μm PTFE filter and equilibrated for 1 h at room temperature, then transferred to disposable microBRAND ultraviolet (UV) cuvettes, and used for DLS measurements.

### Cryogenic Transmission Electron Microscopy (Cryo‐TEM)

Perforated carbon film‐covered microscopical 200 mesh grids (R1/4 batch of Quantifoil, MicroTools GmbH, Jena, Germany) were cleaned with chloroform and hydrophilized by 60 s glow discharging at 10 mA in a Safematic CCU‐010 device (safematic GmbH, Zizers, Switzerland). Subsequently, 4 μl aliquots of the sample solution were applied to the grids. The samples were vitrified by automatic blotting and plunge freezing with a FEI Vitrobot Mark IV (Thermo Fisher Scientific Inc., Waltham, Massachusetts, USA) using liquid ethane as cryogen. The vitrified specimens were transferred to the autoloader of a FEI TALOS ARCTICA electron microscope (Thermo Fisher Scientific Inc., Waltham, Massachusetts, USA). This microscope is equipped with a high‐brightness field‐emission gun (XFEG) operated at an acceleration voltage of 200 kV. Micrographs were acquired on a FEI Falcon 3 direct electron detector (Thermo Fisher Scientific Inc., Waltham, Massachusetts, a 100 μm objective aperture.

### Cytotoxicity

All experiments were conducted following the German genetic engineering laws and German biosafety guidelines in the laboratory (S2). For determining the cell viability a CCK‐8 Kit was used following the manufacturer's instructions. A‐549 cells were cultivated in DMEM‐medium. For cell viability measurements both cell lines were seeded in a 96‐well plate at a density of 5×104 cells/mL in DMEM‐Medium (90.0 μL/well). The seeded cells were incubated over night at 37 °C and 5 % CO2. A 1 : 1 dilution series of each compound was prepared starting with a stock concentration of 12.5 mg/mL. 10.0 μL/well of each compound was applied to the plate including positive (1 % SDS) and negative (Medium, 10 % DPBS) controls. The cells with compound were incubated for another 24 h at 37 °C and 5 % CO2. After 24 h of incubation 10.0 μL/well of CCK‐8 solution was added. After 3 h of incubation at 37 °C and 5 % CO2 the absorbance was measured (450 nm/650 nm) using a Tecan SPARK Plate Reader. All measurement were performed with three technical and one biological repeat. The cell viability was calculated by setting the negative control to 100 % using the Excel software. All Graphs were plotted using GraphPad Prism 6.

### Nile red and Nimodipine Encapsulation and Quantification

The encapsulation and quantification study of the synthesized amphiphiles was done by using hydrophobic dye/drug (Nile red and Nimodipine) through the thin film method by UV‐visible spectral measurement. The dye/drug was solubilized at a concentration of 5 mg mL^−1^ for all the amphiphiles using 0.20 mg of Nile red and 1 mg of Nimodipine. The required amount of dye/drug was taken, dissolved in THF and allowed to evaporate uniformly to form a thin film, followed by the addition of 1 mL aqueous solution of amphiphile. It was stirred at room temperature for 24 h and the non‐encapsulated dye/drug was then removed by filtering, slowly through 0.45 μm PTFE filter. For the quantification of encapsulated dye/drug, the encapsulated samples were lyophilized and redissolved in anhydrous methanol. The absorbance (200–800 nm) was recorded on UV‐Vis spectrophotometer and fluorescence measurement (450–800 nm) was performed on Carry Eclipse fluorescence spectrophotometer with slit width of 5 nm and excitation wavelength of 550 nm for Nile red and 240 nm for Nimodipine. Furthermore, Origin 8 software was used for data analysis.

### Simulation Studies

Molecular dynamics (MD) simulations of drug encapsulation were conducted by inserting 50 amphiphiles (C12−G1 and Py−G1) and 50 drug molecules (Nimodipine and Nile Red) into water, maintaining solution concentrations below 0.4 mol/L. The simulations were performed in cubic simulation boxes with final dimensions of approximately 8 nm, applying periodic boundary conditions in all directions. All simulations were carried out using GROMACS 2024.1, with force field parameters generated by LigParGen (available at https://zarbi.chem.yale.edu/ligpargen) based on OPLS parameters. Atomic partial charges were calculated on DFT‐optimized molecules (B3LYP/6‐31G*) using the CHELPG method.

After an initial energy minimization step using the steepest descent algorithm, NPT simulations were performed with a time step of 2 fs for 300 ns under conditions of 1 atm pressure (using the Parrinello‐Rahman barostat) and 298 K temperature (controlled by the V‐rescale thermostat). The cutoff for non‐bonded interactions was set to 1.2 nm, and long‐range electrostatics were handled using the PME method with a pme_order of 4. Constraints were applied to hydrogen bonds. Snapshots shown in Figure 7 were taken at the end of the 300 ns simulation, and aggregate visualization was performed using VMD (DOI: 10.1016/0263‐7855(96)00018‐5).

### Cellular Uptake Studies

The Cellular uptake of C12−G1 loaded with Nile red in A‐549 cancer cell line was monitored using confocal laser scanning microscopy (cLSM). The cells were routinely cultivated. For cLSM, 270 μL of cells in DMEM with a cell density of 5×10 ^4^ cells/mL were seeded in each well of an 8‐ well μ‐slide. After incubation over night at 37 °C and 5.00 % CO_2_ 30.0 μL of Nile red loaded compound were added at a final test concentration of 1.25 mg/mL. As a positive control 30.0 μL of Nile red in DMSO at a final test concentration of 0.0002 mg/mL was added. After incubation for 6 h the cell nuclei was stained with 1.00 μg/mL Hoechst 33342 (Life Technologies GmbH, Darmstadt, Germany). The confocal images were taken by using an inverted confocal laser scanning microscope Leica DMI6000CSB SP8 (Leica, Wetzlar, Germany) with an 63x/1.4 HC PL APO CS2 oil immersion objective using the manufacture given LAS X software. The Images were analysed using the Fiji version of ImageJ 1.54j. The images were analysed by enhancing the contrast. Thereby the number of saturated pixels was set to 0.50 % for all images. A scale bar was added to all images.

### Release Study

Since the synthesized amphiphiles contains ester bond, the enzymatic cleavage of this bond was investigated to release the encapsulated Nile Red. Enzyme mediated cleavage was performed with a general de‐esterification method using Candida Antarctica Lipase b. C12−G1 has been taken to study the release behaviour of Nile Red. Initially, Nile Red was encapsulated in C12−G1 by following the same protocol as discussed in the dye/drug encapsulation section in main manuscript, after removing all the insoluble dye using 0.45‐micron RC membranes, a few drops of n‐butanol were added and it was stirred at 37 °C. The fluorescence intensity was measured time to time to check the release behaviour of the nano‐carrier. A complete release of Nile Red was observed after 12 h.

### Synthetic Procedure

A series of four novel dendritic amphiphiles have been synthesized as shown in Scheme 1. All of the amphiphiles were well characterized by their physiochemical and spectroscopic data. Total four of the Amphiphile were synthesized using G1−OH as hydrophilic and four different hydrophobic segments.

### Synthesis of C12−G1 Amphiphiles

In a round bottom flask, G1‐OH dendron (1 eq, 0.5 g, 1.56 mmol) and 10 ml of dry CH_2_Cl_2_ were taken. To this solution EDC.HCl (1 eq, 0.3 g, 1.55 mmol) and DMAP (1 eq, 0.19 g, 1.55 mmol) were added. The reaction mixture was stirred at room temperature, followed by dodecanoic acid (1.3 eq, 0.4 g, 1.99 mmol). The resultant mixture was stirred for 24 h at room temperature. The progress of the reaction was monitored by TLC (Petroleum ether: Ethyl acetate, 70 : 30). After the completion of the reaction, the solvent was evaporated under reduced pressure, and the reaction mixture was extracted with saturated NaHCO_3_ solution and dichloromethane. The combined organic layer was dried over Na_2_SO_4_ and the solvent was removed by rotary evaporation. The crude product was purified by column chromatography. The resultant product was taken in round bottom flask and add Dowex‐50 in methanol to obtain the final amphiphile C12−G1.

Yield‐ 95 %, ^1^H NMR (400 MHz, CD_3_OD, ppm): 5.11–5.07 (1H, m, −CH−G1), 3.70–3.70 (2H, m, −CH−G1), 3.58–3.39 (m, 12H, −CH_2_−G1), 2.28 (2H, t, *J*=8 Hz, – CH_2_, Alkyl chain), 1.58‐1.53 (2H, m, – CH_2_, Alkyl chain), 1.29‐1.24 (18H, m,– CH_2_, Alkyl chain), 0.84 (3H, t, *J*=4 Hz, −CH_3_ Alkyl chain); ^13^C NMR (151 MHz, CHLOROFORM‐*D*) δ 177.68, 76.51, 75.44, 74.79, 73.74, 67.02, 52.47, 37.82, 35.70, 33.36, 33.24, 32.79, 28.67, 26.36, 17.06.

### Synthesis of C8−G1 Amphiphiles

In a round bottom flask, G1−OH dendron (1 eq, 0.5 g, 1.56 mmol) and 10 ml of dry CH_2_Cl_2_ were taken. To this solution EDC.HCl (1 eq,0.3 g, 1.55 mmol) and DMAP (1 eq, 0.19 g, 1.55 mmol) were added. The reaction mixture was stirred at room temperature, followed by octanoic acid (1.3 eq,0.3 g, 2.01 mmol). The resultant mixture was stirred for 24 h at room temperature. The progress of the reaction was monitored by TLC (Petroleum ether: Ethyl acetate, 70 : 30). After the completion of the reaction, the solvent was evaporated under reduced pressure, and the reaction mixture was extracted with saturated NaHCO_3_ solution and dichloromethane. The combined organic layer was dried over Na_2_SO_4_ and the solvent was removed by rotary evaporation. The crude product was purified by column chromatography. Further, the resultant product was taken in round bottom flask and add Dowex‐50 (20 wt %) in methanol to obtain the final acetal deprotected amphiphile C8−G1.

Yield‐ 85 %, ^1^H NMR (400 MHz, CD_3_OD, ppm): 5.10–5.06 (1H, m, ‐CH−G1), 3.69–3.65 (2H, m, −CH−G1), 3.57–3.43 (m, 12H, −CH_2_−G1), 2.28 (2H, t, *J*=8 Hz, – CH_2_, Alkyl chain), 1.58–1.52 (2H, m, – CH_2_, Alkyl chain), 1.28–1.24 (10H, m, – CH_2_, Alkyl chain), 0.84 (3H, t, *J*=4 Hz, −CH_3_ Alkyl chain); ^13^C NMR (151 MHz, CHLOROFORM‐*D*) δ 177.68, 76.51, 75.45, 74.79, 73.77, 67.05, 37.82, 35.48, 32.74, 28.67, 26.29, 17.03.

### Synthesis of Nap−G1 Amphiphiles

In a round bottom flask, G1−OH dendron (1 eq, 0.5 g, 1.56 mmol) and 10 ml of dry CH_2_Cl_2_ were taken. To this solution EDC.HCl(1 eq, 0.3 g, 1.55 mmol) and DMAP (1 eq, 0.19 g, 1.55 mmol) were added. The reaction mixture was stirred at room temperature, followed by 2‐(naphthalen‐1‐yl) acetic acid (1.3 eq, 0.378 g, 2.03 mmol). The resultant mixture was stirred for 24 h at room temperature. The progress of the reaction was monitored by TLC (Petroleum ether: Ethyl acetate, 70 : 30). After the completion of the reaction, the solvent was evaporated under reduced pressure, and the reaction mixture was extracted with saturated NaHCO_3_ solution and dichloromethane. The combined organic layer was dried over Na_2_SO_4_ and the solvent was removed by rotary evaporation. The crude product was purified by column chromatography. The resultant product was taken in round bottom flask and add Dowex‐50 in methanol to obtain the final amphiphile Nap‐G1.

Yield‐ 88 %, 1H NMR (400 MHz, CD_3_OD, ppm): 7.94–7.72 (3H, m, −CH aromatic ring), 7.48–7.35 5.09 (4H, m, −CH aromatic ring), 5.11–5.08 (1H, m, −CH−G1), 3.61–3.56 (2H, m, −CH−G1), 3.53–3.26 (m, 12H, −CH_2_−G1); ^13^C NMR (151 MHz, METHANOL‐*D*_4_) δ 173.08, 135.29, 133.51, 132.01, 129.69, 129.17, 129.03, 127.34, 126.80, 126.52, 124.97, 73.83, 73.63, 72.12, 71.00, 64.35, 54.80, 39.84.

### Synthesis of Py−G1 Amphiphiles

In a round bottom flask, G1‐OH dendron (1eq, 0.5 g, 1.56 mmol) and 10 ml of dry CH_2_Cl_2_ were taken. To this solution EDC.HCl (1 eq, 0.3 g, 1.55 mmol) and DMAP (1 eq, 0.19 g, 1.55 mmol) were added. The reaction mixture was stirred at room temperature, followed by 2‐(4,5‐dihydropyren‐2‐yl) acetic acid (1.3 eq, 0.528 g, 2.03 mmol). The resultant mixture was stirred for 24 h at room temperature. The progress of the reaction was monitored by TLC (Petroleum ether: Ethyl acetate, 70 : 30). After the completion of the reaction, the solvent was evaporated under reduced pressure, and the reaction mixture was extracted with saturated NaHCO_3_ solution and dichloromethane. The combined organic layer was dried over Na_2_SO_4_ and the solvent was removed by rotary evaporation. The crude product was purified by column chromatography. The resultant product was taken in round bottom flask and add Dowex‐50 in methanol to obtain the final amphiphile Py−G1.

Yield‐ 92 %, ^1^H NMR (400 MHz, CD_3_OD, ppm): 8.24–8.11 (m, 5H, −CH aromatic ring), 8.02–7.91 (4H, m, −CH aromatic ring), 5.16–5.11 (1H, m, −CH), 3.60–3.52 (6H, m, −CH_2_ and −CH−G1), 3.39–3.28 (8H, m, −CH_2_ and −CH−G1): ^13^C NMR (100 MHz, CD_3_OD, ppm) δ 184.57, 127.47, 126.46, 124.36, 124.15, 123.88, 70.84, 69.78, 66.95, 66.72, 63.89, 63.64, 38.53, 38.27.

## Conflict of Interests

The authors declare no conflict of interest.

## Supporting information

As a service to our authors and readers, this journal provides supporting information supplied by the authors. Such materials are peer reviewed and may be re‐organized for online delivery, but are not copy‐edited or typeset. Technical support issues arising from supporting information (other than missing files) should be addressed to the authors.

Supporting Information

## Data Availability

The data that support the findings of this study are available in the supplementary material of this article.
