# Supplementary material for: Synthesis of Dendritic Oligo‐Glycerol Amphiphiles with Different Hydrophobic Segments to Improve their Performance as Nanocarriers
Source: ChemistryOpen. 2025 Feb 10;14(8):e202400448. doi: 10.1002/open.202400448 (PMC12368886; doi:10.1002/open.202400448)
Supplement: Supplementary file 1 — Supporting Information [file OPEN-14-e202400448-s001.pdf]

ChemistryOpen

Supporting Information

Synthesis of Dendritic Oligo-Glycerol Amphiphiles with Different Hydrophobic Segments to Improve their Performance as Nanocarriers

Pooja Kumari, Christian Zoister, Natalie Hanheiser, Hesam Makki, Boris Schade, Mathias Dimde, Katharina Achazi, Sumit Kumar,* Rainer Haag,* and Abhishek K. Singh*

SUPPLEMENTARY INFORMATION

Synthesis of Dendritic Oligo-glycerol Amphiphiles with Different Hydrophobic Segments to Improve their Performance as Nanocarriers

Pooja Kumari[a,b], Christian Zoister[b], Natalie Hanheiser[b], Hesam Makki[d], Boris Schade[c], Mathias Dimde[c], Katharina Achazi[b], Sumit Kumar[*a], Rainer Haag[*b], Abhishek K. Singh[*b]

[a]*Department of Chemistry, Deenbandhu Chhoturam University of Science and Technology, 131039, Murthal, Sonipat, India*

[b]*Institut für Chemie und Biochemie Organische Chemie, Freie Universität Berlin, Takustr. 3, 14195 Berlin, Germany*

[c]*Institut fu¨r Chemie und Biochemie, Forschungszentrum fu¨r Elektronenmikroskopie, Freie Universita¨t Berlin, Fabeckstraße 36a, 14195 Berlin, Germany*

[d]*Department of Chemistry and Materials Innovation Factory, University of Liverpool, L69 7ZD Liverpool, U.K*

Table of Contents

1. Experimental Sections

1.1 Materials

1.2 Instrumentation

1.3 Synthetic Procedure

2. Figures

Figure S1. ^{1}H and ^{13}C NMR spectra of amphiphile C12-G1.

Figure S2. ^{1}H and ^{13}C NMR spectra of amphiphile C8-G1.

Figure S3. ^{1}H and ^{13}C NMR spectra of amphiphile Nap-G1.

Figure S4. ^{1}H and ^{13}C NMR spectra of amphiphile Py-G1.

Figure S5. HRMS of amphiphile C12-G1

Figure S6. HRMS of amphiphile C8-G1

Figure S7. HRMS of amphiphile Nap-G1

Figure S8. HRMS of amphiphile Py-G1

Figure S9. Cytotoxicity of the synthesized amphiphiles.

Figure S10. Intensity and number size distributions of synthesized amphiphiles.

Figure S10. UV Absorbance spectra of Nile red for amphiphiles in aq. solution

Figure S11. UV Absorbance spectra of Nimodipine for amphiphiles in aq. solutions.

1. Experimental Section

1.1 Materials

All the chemicals and solvents used were procured from Sigma-Aldrich Chemicals, USA. Immobilized Candida antarctica lipase (Novozym 435) was obtained from Novo Nordisk A/S Denmark. For the encapsulation studies the dyes/drugs used were bought from Sigma-Aldrich Chemicals, USA with determined purity. To monitor the reaction progress, Pre-coated TLC plate (Merck silica gel 60F254) was used and $KMnO_4$ staining solution was used to visualize the spots on TLC plate. Silica gel (60-120 mesh) was used for column chromatography. Millipore water was used for preparation of samples for their physico-chemical characterization and transport studies. For the cell toxicity, 96-well flat plate transparent (Sarstedt 96 Flat Transparent Cat. No.: 83.3924), DMEM (1X) + GlutaMAX^{TM} (Gibco, Ref. No.: 31966-021) + 10% fetal bovine serum (FBS) (Sigma-Aldrich, Co., Cat. No.: F7524) + 1% penicillin-streptomycin (PS) (Sigma-Aldrich, Co., Cat. No.: P4333), Phosphate buffer solution (DPBS, w/o Calcium, w/o Magnesium) (pH= 7.2) (PAN-Biotech, Cat. No.: P04-36500) , Cell lines: both cell lines were obtained from Leibniz Institute DSMZ- German Collection of Microorganisms and Cell Cultures GmbH, A-549 ACC 107, CCK-8 Kit (Hycultec, Art. No.: HY-K0301), Tecan SPARK Plate Reader (Tecan Austria GmbH, Ref. No.: 30086376) have been used. And for cellular uptake µ-slides 8 Well ibidi, 1x µ-Slide 8 Well ibiTreat (Cat.No: 80806), Hoechst 33342 (Life Technologies GmbH, Darmstadt, Germany), c = 10 mg/mL stock solution in DMSO (stored at -20 °C, protected from light), Nile Red, c= 0.002 mg/mL stock solution in DMSO (stored at 4 °C, protected from light), Confocal laser scanning microscope Leica DMI6000CSB SP8 (Leica, Wetzlar, Germany) have been used.

1.2.2 Critical aggregation concentration (CAC) measurements

Synthesized amphiphiles' critical aggregation concentration was studied by fluorescence measurement technique using 'Nile red' as a model dye. A stock solution of the dye was prepared in THF at the concentration 1 mg mL^{-1}. To form thin layer, 10 µL of the stock solution was added to each empty vial followed by complete evaporation of THF. The stock solutions of amphiphiles (1 mM) were prepared in Milli-Q water. To achieve different concentrations of the amphiphiles, two-fold serial dilution of the stock solutions was done and the solution was then transferred to the vial having thin film of the dye and stirred overnight. Polytetrafluoroethylene (PTFE) filter (0.45 µm) was used to remove the non-encapsulated dye from the solutions with subsequent fluorescence measurements using Cary Eclipse fluorescence spectrophotometer. The plot of fluorescence intensity maxima values against log [amphiphile concentration] for different samples was used for calculating the CAC value.

1.2.3 Dynamic Light Scattering (DLS)

Malvern Zeta sizer Nano ZS analyzer integrated with 4 mW He-Ne laser, λ= 633 nm, using backscattering detection (scattering angle θ = 173°) with an avalanche photodiode detector, was used for determining the size of nanostructures (micelles/aggregates) formed by the supramolecular organization of amphiphiles in the aqueous solution (Milli-Q water) at a concentration of 5 mg mL^{-1}. The samples were then further allowed to mix at 25 °C for 20 h with vigorous stirring. The obtained solutions were then filtered through 0.45 µm PTFE filter and equilibrated for 1 h at room temperature, then transferred to disposable microBRAND ultraviolet (UV) cuvettes, and used for DLS measurements.

1.2.4 Cryogenic transmission electron microscopy (Cryo-TEM)

Perforated carbon film-covered microscopical 200 mesh grid (R1/4 batch of Quantifoil, Micro Tools GmbH, Jena, Germany) were cleaned with chloroform and hydrophilised by 60 s glow discharging at 8 W in a BAL-TEC MED 020 device (Leice Microsystem, Wetzlar, Germany) before 5 µL aliquots of the sample solution (5 mg mL-1) were applied to the grids. The samples were automatically blotted and vitrified with FEI Vitrobot Mark IV (Thermo Fisher Scientific Inc., Waltham, Massachusetts, USA) using liquid ethane as cryogen. Cryo-TEM measurements were carried out on a Tecnai F20 TEM (FEI Company, Oregon) equipped with a field emission gun (FEG) at an acceleration voltage of 160 kV using a Gatan cryo holder at 94 K sample temperature. By using the microscope's low-dose protocol, the micrographs were recorded with a FEI Eagle 4k X 4k CCD camera in twofold binning mode.

1.2.5 Cytotoxicity

All experiments were conducted following the German genetic engineering laws and German biosafety guidelines in the laboratory (S2). For determining the cell viability a CCK-8 Kit was used following the manufacturer's instructions. A-549 cells were cultivated in DMEM-medium. For cell viability measurements both cell lines were seeded in a 96-well plate at a density of 5x104 cells/mL in DMEM-Medium (90.0 µL/well). The seeded cells were incubated over night at 37°C and 5% CO2. A 1:1 dilution series of each compound was prepared starting with a stock concentration of 12.5 mg/mL. 10.0 µL/well of each compound was applied to the plate including positive (1% SDS) and negative (Medium, 10% DPBS) controls. The cells with compound were incubated for another 24h at 37°C and 5% CO2. After 24h of incubation 10.0 µL/well of CCK-8 solution was added. After 3h of incubation at 37°C and 5% CO2 the absorbance was measured (450nm/650nm) using a Tecan SPARK Plate Reader. All measurement were performed with three technical and one biological repeat. The cell viability was calculated by setting the negative control to 100% using the Excel software. All Graphs were plotted using GraphPad Prism 6.

1.2.6 Nile red and Nimodipine encapsulation and quantification

The encapsulation and quantification study of the synthesized amphiphiles was done by using hydrophobic dye/drug (Nile red and Nimodipine) through the thin film method by UV-visible spectral measurement. The dye/drug was solubilized at a concentration of 5 mg mL^{-1} for all the amphiphiles using 0.20 mg of Nile red and 1 mg of Nimodipine. The required amount of dye/drug was taken, dissolved in THF and allowed to evaporate uniformly to form a thin film, followed by the addition of 1 mL aqueous solution of amphiphile. It was stirred at room

temperature for 24 h and the non-encapsulated dye/drug was then removed by filtering, slowly through 0.45 µm PTFE filter. For the quantification of encapsulated dye/drug, the encapsulated samples were lyophilized and redissolved in anhydrous methanol. The absorbance (200-800 nm) was recorded on UV-Vis spectrophotometer and fluorescence measurement (450-800 nm) was performed on Carry Eclipse fluorescence spectrophotometer with slit width of 5 nm and excitation wavelength of 550 nm for Nile red and 240 nm for Nimodipine. Furthermore, Origin 8 software was used for data analysis.

1.2.7 Simulation studies

Molecular dynamics (MD) simulations of drug encapsulation were conducted by inserting 50 amphiphiles (C12-G1 and Py-G1) and 50 drug molecules (Nimodipine and Nile Red) into water, maintaining solution concentrations below 0.4 mol/L. The simulations were performed in cubic simulation boxes with final dimensions of approximately 8 nm, applying periodic boundary conditions in all directions. All simulations were carried out using GROMACS 2024.1, with force field parameters generated by LigParGen (available at https://zarbi.chem.yale.edu/ligpargen) based on OPLS parameters. Atomic partial charges were calculated on DFT-optimized molecules (B3LYP/6-31G*) using the CHELPG method.

After an initial energy minimization step using the steepest descent algorithm, NPT simulations were performed with a time step of 2 fs for 300 ns under conditions of 1 atm pressure (using the Parrinello-Rahman barostat) and 298 K temperature (controlled by the V-rescale thermostat). The cutoff for non-bonded interactions was set to 1.2 nm, and long-range electrostatics were handled using the PME method with a pme_order of 4. Constraints were applied to hydrogen bonds. Snapshots shown in Figure 7 were taken at the end of the 300 ns simulation, and aggregate visualization was performed using VMD (DOI: 10.1016/0263-7855(96)00018-5).

1.2.8 Cellular Uptake Studies

The Cellular uptake of C12-G1 loaded with Nile red in A-549 cancer cell line was monitored using confocal laser scanning microscopy (cLSM). The cells were routinely cultivated. For cLSM, 270 µL of cells in DMM with a cell density of 5 x10 ^{4} cells/mL were seeded in each well of an 8- well µ-slide. After incubation over night at 37°C and 5.00 % CO_2 30.0 µL of Nile red loaded compound were added at a final test concentration of 1.25 mg/mL. As a positive control 30.0 µL of Nile red in DMSO at a final test concentration of 0.0002 mg/mL was added. After incubation for 6 h the cell nuclei was stained with 1.00 µg/mL Hoechst 33342 (Life Technologies GmbH, Darmstadt, Germany). The confocal images were taken by using an inverted confocal laser scanning microscope Leica DMI6000CSB SP8 (Leica, Wetzlar, Germany) with an 63x/1.4 HC PL APO CS2 oil immersion objective using the manufacture given LAS X software. The Images were analysed using the Fiji version of ImageJ 1.54j[1]. The images were analysed by enhancing the contrast. Thereby the number of saturated pixels was set to 0.50 % for all images. A scale bar was added to all images.

1.2.9 Release study

Since the synthesized amphiphiles contains ester bond, the enzymatic cleavage of this bond was investigated to release the encapsulated Nile Red. Enzyme mediated cleavage was performed with a general de-esterification method using Candida Antarctica Lipase b. C12-G1 has been taken to study the release behaviour of Nile Red. Initially, Nile Red was encapsulated in C12-G1 by following the same protocol as discussed in the dye/drug encapsulation section in main manuscript, after removing all the insoluble dye using 0.45-micron RC membranes, a few drops of n-butanol were added and it was stirred at 37^oC. The fluorescence intensity was measured time to time to check the release behaviour of the nano-carrier. A complete release of Nile Red was observed after 12h.

1.3 Synthetic Procedure

A series of four novel dendritic amphiphiles have been synthesized as shown in Scheme 1. All of the amphiphiles were well characterized by their physiochemical and spectroscopic data. Total four of the Amphiphile were synthesized using G1-OH as hydrophilic and four different hydrophobic segments.

1. **Synthesis of C12-G1 Amphiphiles**

In a round bottom flask, G1-OH dendron (1eq, 0.5g, 1.56mmol) and 10 ml of dry CH_2Cl_2 were taken. To this solution EDC.HCl (1eq, 0.3g, 1.55mmol) and DMAP (1eq, 0.19g, 1.55mmol) were added. The reaction mixture was stirred at room temperature, followed by dodecanoic acid (1.3eq, 0.4g, 1.99mmol). The resultant mixture was stirred for 24h at room temperature. The progress of the reaction was monitored by TLC (Petroleum ether: Ethyl acetate, 70:30). After the completion of the reaction, the solvent was evaporated under reduced pressure, and the reaction mixture was extracted with saturated $NaHCO_3$ solution and dichloromethane. The combined organic layer was dried over Na_2SO_4 and the solvent was removed by rotary evaporation. The crude product was purified by column chromatography. The resultant product was taken in round bottom flask and add Dowex-50 in methanol to obtain the final amphiphile C12-G1.

Yield- 95%, ^{1}H NMR (400 MHz, CD_3OD, ppm): 5.11-5.07 (1H, m, -CH-G1), 3.70-3.70 (2H, m, -CH-G1), 3.58-3.39 (m, 12H,-CH_2-G1), 2.28 (2H, t, *J*=8Hz, - CH_2, Alkyl chain), 1.58-1.53 (2H, m, – CH_2, Alkyl chain), 1.29-1.24 (18H, m,- CH_2, Alkyl chain), 0.84 (3H, t, *J*=4Hz, -CH_3 Alkyl chain); ^{13}C NMR (151 MHz, CHLOROFORM-*D*) δ 177.68, 76.51, 75.44, 74.79, 73.74, 67.02, 52.47, 37.82, 35.70, 33.36, 33.24, 32.79, 28.67, 26.36, 17.06.

2. **Synthesis of C8-G1 Amphiphiles**

In a round bottom flask, G1-OH dendron (1eq, 0.5g, 1.56mmol) and 10 ml of dry CH_2Cl_2 were taken. To this solution EDC.HCl (1eq,0.3g, 1.55mmol) and DMAP (1eq, 0.19g, 1.55mmol) were added. The reaction mixture was stirred at room temperature, followed by octanoic acid (1.3eq,0.3g, 2.01mmol). The resultant mixture was stirred for 24h at room temperature. The progress of the reaction was monitored by TLC (Hexane: Ethyl acetate,

70:30). After the completion of the reaction, the solvent was evaporated under reduced pressure, and the reaction mixture was extracted with saturated $NaHCO_3$ solution and dichloromethane. The combined organic layer was dried over Na_2SO_4 and the solvent was removed by rotary evaporation. The crude product was purified by column chromatography. Further, the resultant product was taken in round bottom flask and add Dowex-50 (20wt%) in methanol to obtain the final acetal deprotected amphiphile C8-G1.

Yield- 85%, ^{1}H NMR (400 MHz, CD_3OD, ppm): 5.10-5.06 (1H, m, -CH-G1), 3.69-3.65 (2H, m, -CH-G1), 3.57-3.43 (m, 12H,-CH_2-G1), 2.28 (2H, t, *J*=8Hz, - CH_2, Alkyl chain), 1.58-1.52 (2H, m, – CH_2, Alkyl chain), 1.28-1.24 (10H, m,- CH_2, Alkyl chain), 0.84 (3H, t, *J*=4Hz, -CH_3 Alkyl chain); ^{13}C NMR (151 MHz, CHLOROFORM-*D*) δ 177.68, 76.51, 75.45, 74.79, 73.77, 67.05, 37.82, 35.48, 32.74, 28.67, 26.29, 17.03.

3. Synthesis of Nap-G1 Amphiphiles

In a round bottom flask, G1-OH dendron (1eq, 0.5g, 1.56mmol) and 10 ml of dry CH_2Cl_2 were taken. To this solution EDC.HCl(1eq, 0.3g, 1.55mmol) and DMAP (1eq, 0.19g, 1.55mmol) were added. The reaction mixture was stirred at room temperature, followed by 2-(naphthalen-1-yl) acetic acid (1.3eq, 0.378g, 2.03mmol). The resultant mixture was stirred for 24h at room temperature. The progress of the reaction was monitored by TLC (Petroleum ether: Ethyl acetate, 70:30). After the completion of the reaction, the solvent was evaporated under reduced pressure, and the reaction mixture was extracted with saturated $NaHCO_3$ solution and dichloromethane. The combined organic layer was dried over Na_2SO_4 and the solvent was removed by rotary evaporation. The crude product was purified by column chromatography. The resultant product was taken in round bottom flask and add Dowex-50 in methanol to obtain the final amphiphile Nap-G1.

Yield- 88%, 1H NMR (400 MHz, CD_3OD, ppm): 7.94-7.72 (3H, m, -CH aromatic ring), 7.48-7.35 5.09 (4H, m, -CH aromatic ring), 5.11-5.08 (1H, m, -CH-G1), 3.61-3.56 (2H, m, -CH-G1), 3.53-3.26 (m, 12H,-CH_2-G1); ^{13}C NMR (151 MHz, METHANOL-D_4) δ 173.08, 135.29, 133.51, 132.01, 129.69, 129.17, 129.03, 127.34, 126.80, 126.52, 124.97, 73.83, 73.63, 72.12, 71.00, 64.35, 54.80, 39.84.

4. Synthesis of Py-G1 Amphiphiles

In a round bottom flask, G1-OH dendron (1eq, 0.5g, 1.56mmol) and 10 ml of dry CH_2Cl_2 were taken. To this solution EDC.HCl (1eq, 0.3g, 1.55mmol) and DMAP (1eq, 0.19g, 1.55mmol) were added. The reaction mixture was stirred at room temperature, followed by 2-(4,5-dihydropyren-2-yl) acetic acid (1.3eq, 0.528g, 2.03mmol). The resultant mixture was stirred for 24h at room temperature. The progress of the reaction was monitored by TLC (Petroleum ether: Ethyl acetate, 70:30). After the completion of the reaction, the solvent was evaporated under reduced pressure, and the reaction mixture was extracted with saturated $NaHCO_3$ solution and dichloromethane. The combined organic layer was dried over Na_2SO_4 and the solvent was removed by rotary evaporation. The crude product

was purified by column chromatography. The resultant product was taken in round bottom flask and add Dowex-50 in methanol to obtain the final amphiphile Py-G1.

Yield- 92%, ^{1}H NMR (400 MHz, CD_3OD, ppm): 8.24-8.11 (m, 5H, -CH aromatic ring), 8.02-7.91 (4H, m, -CH aromatic ring), 5.16-5.11 (1H, m, -CH), 3.60-3.52 (6H, m, -CH_2 and -CH-G1), 3.39-3.28 (8H, m, -CH_2 and -CH-G1): ^{13}C NMR (100 MHz, CD_3OD, ppm) δ 184.57, 127.47, 126.46, 124.36, 124.15, 123.88, 70.84, 69.78, 66.95, 66.72, 63.89, 63.64, 38.53, 38.27.

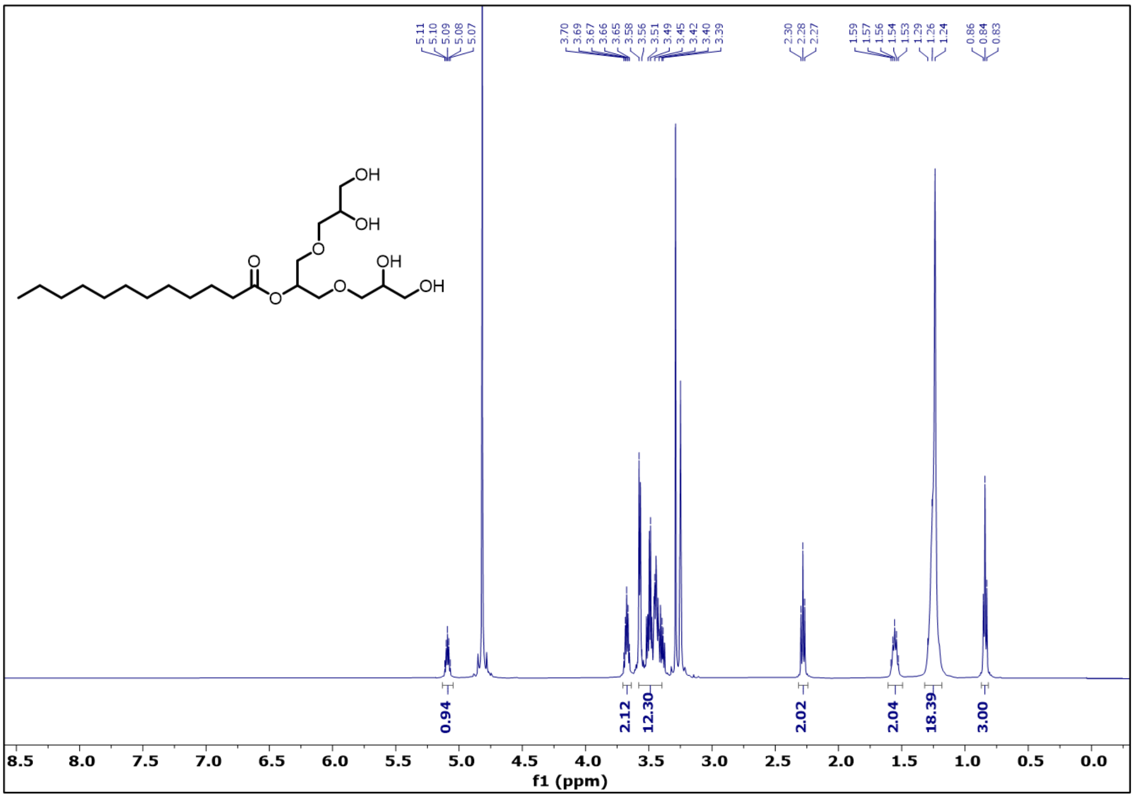

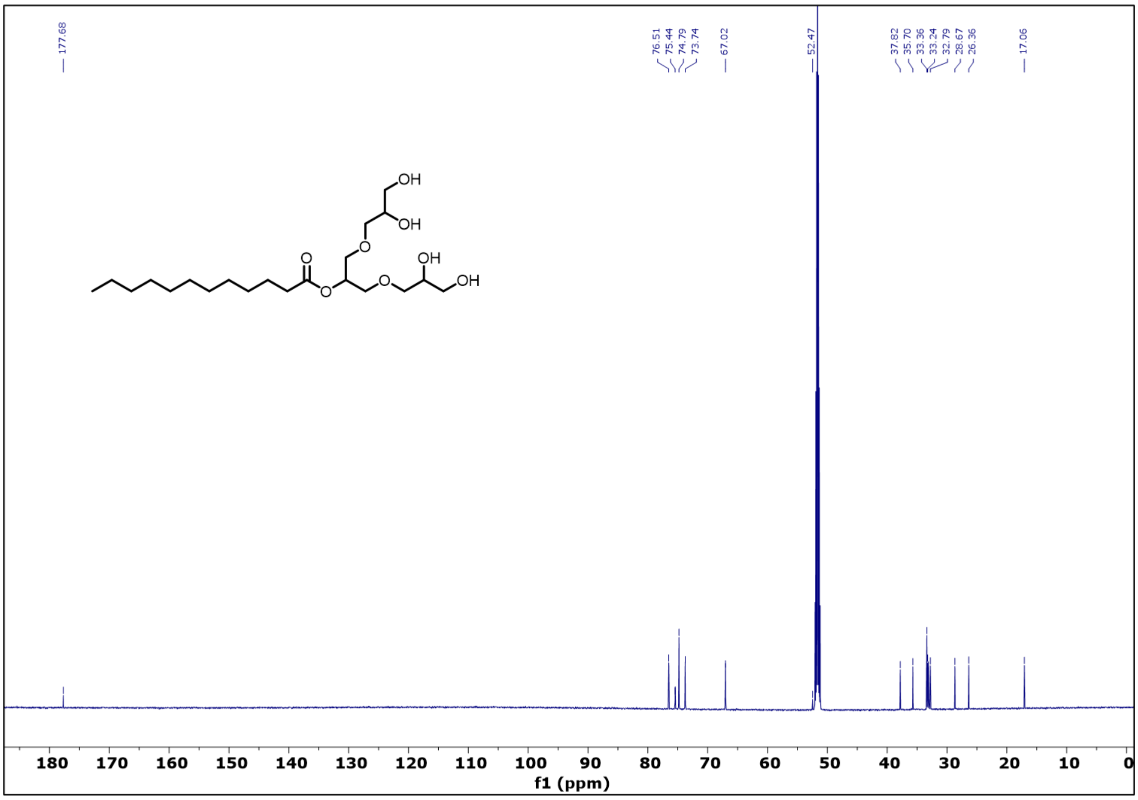

Figure S1. ^{1}H and ^{13}C NMR spectra of amphiphile C12-G1.

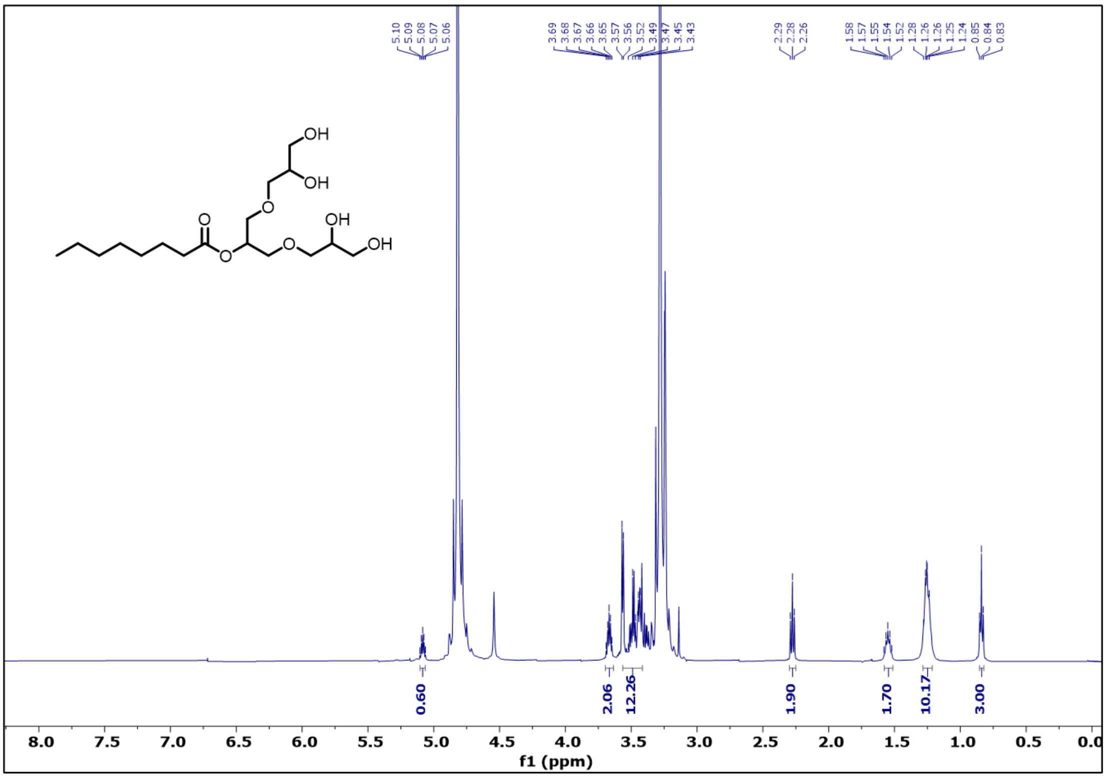

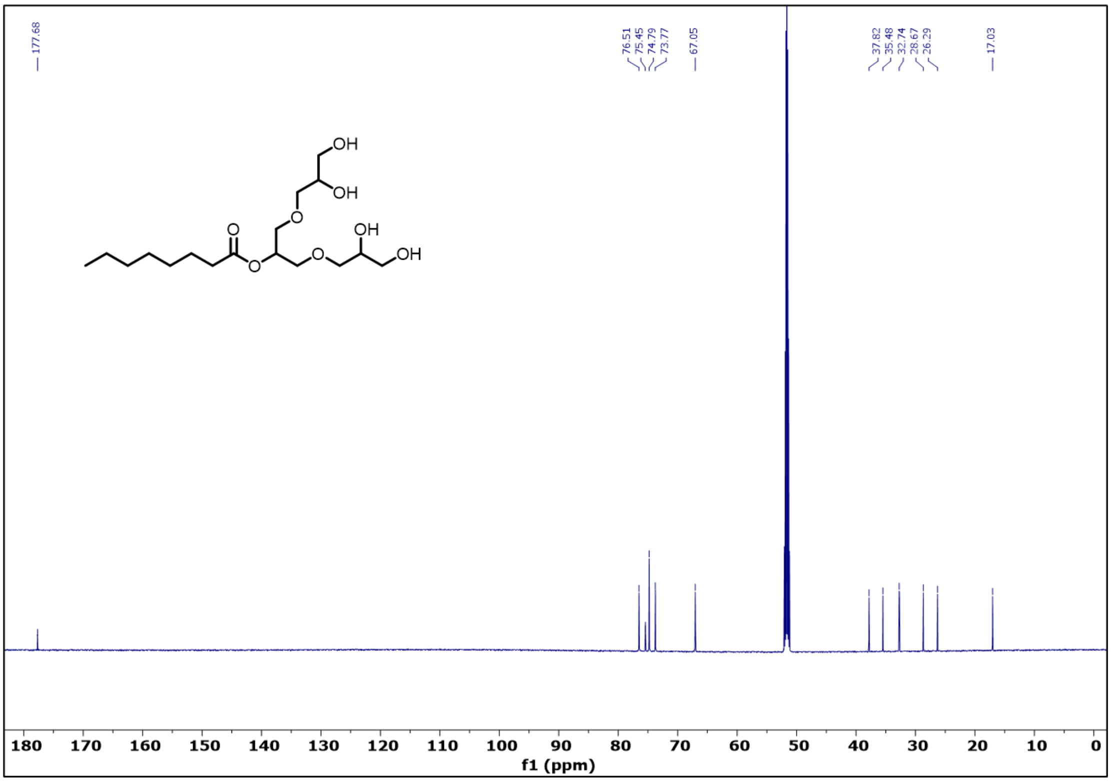

Figure S2. 1H NMR spectra of amphiphile C8-G1.

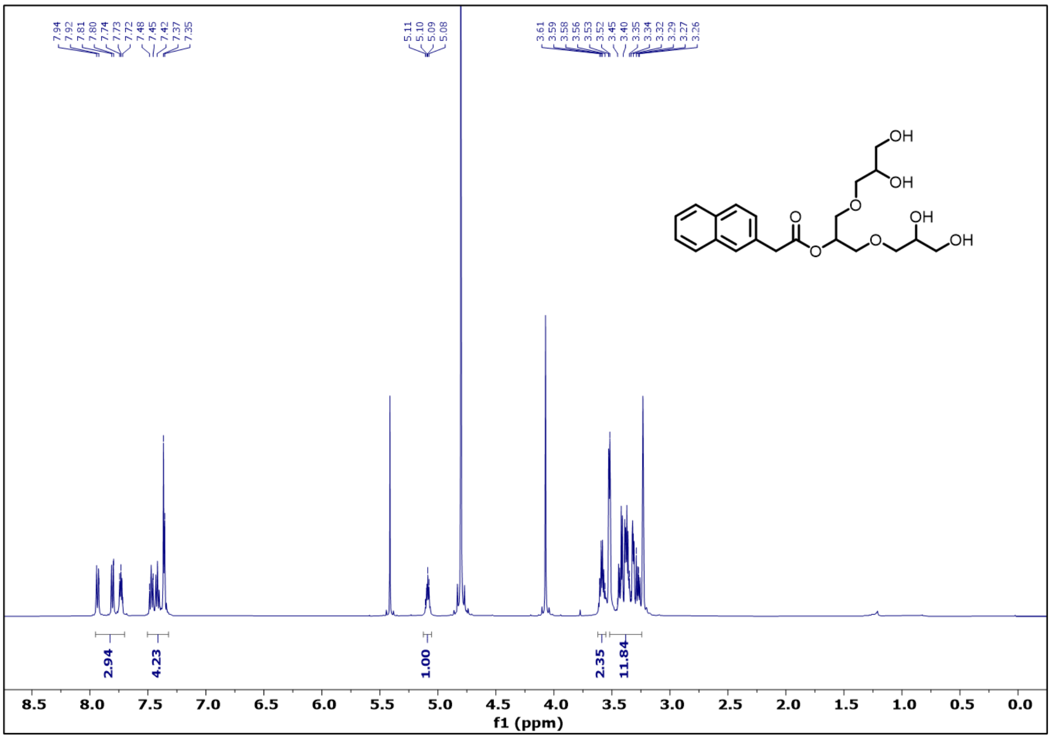

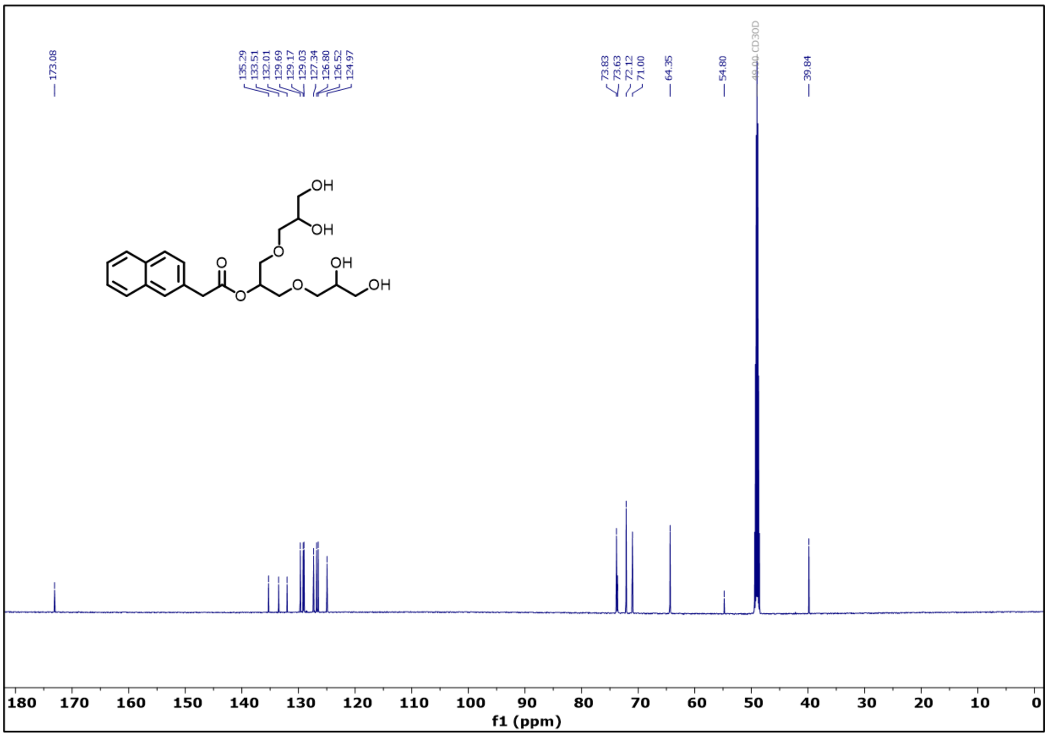

Figure S3. ^{1}H and ^{13}C NMR spectra of amphiphile Nap-G1.

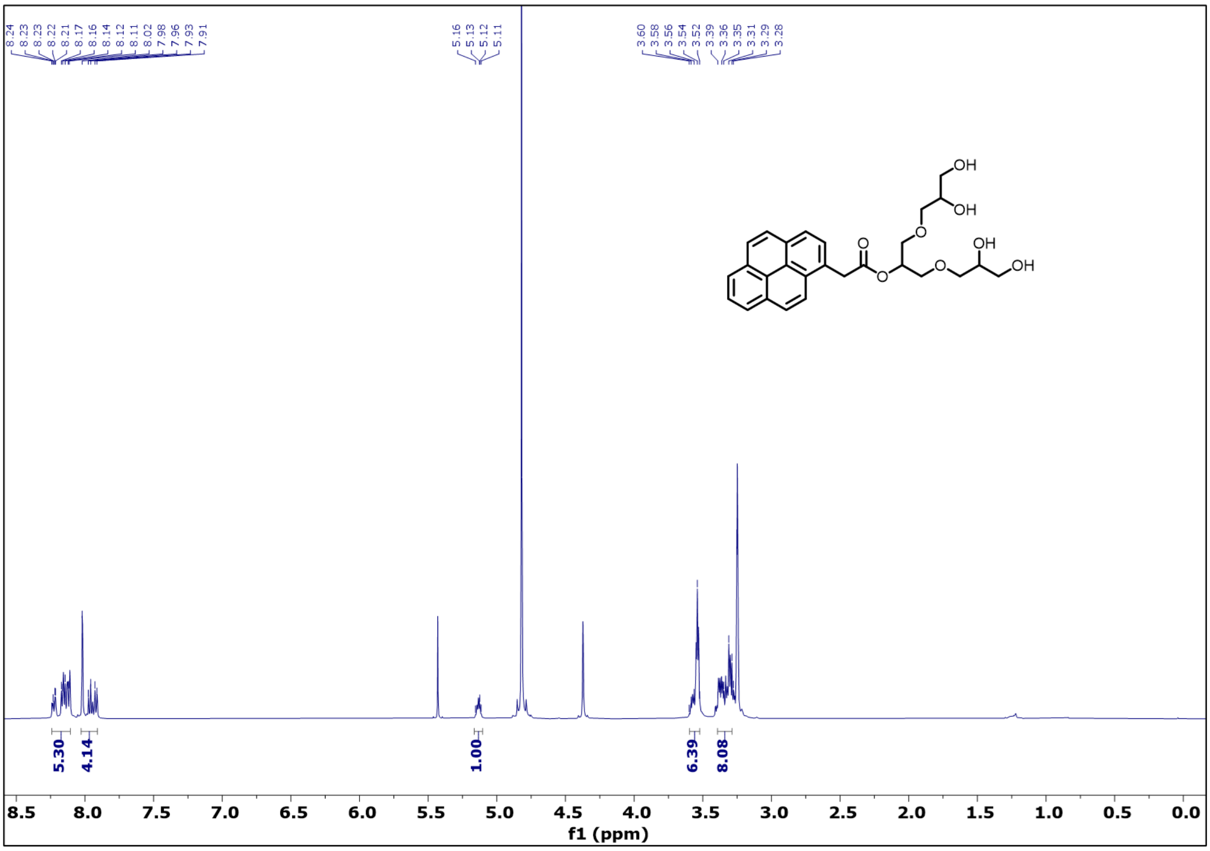

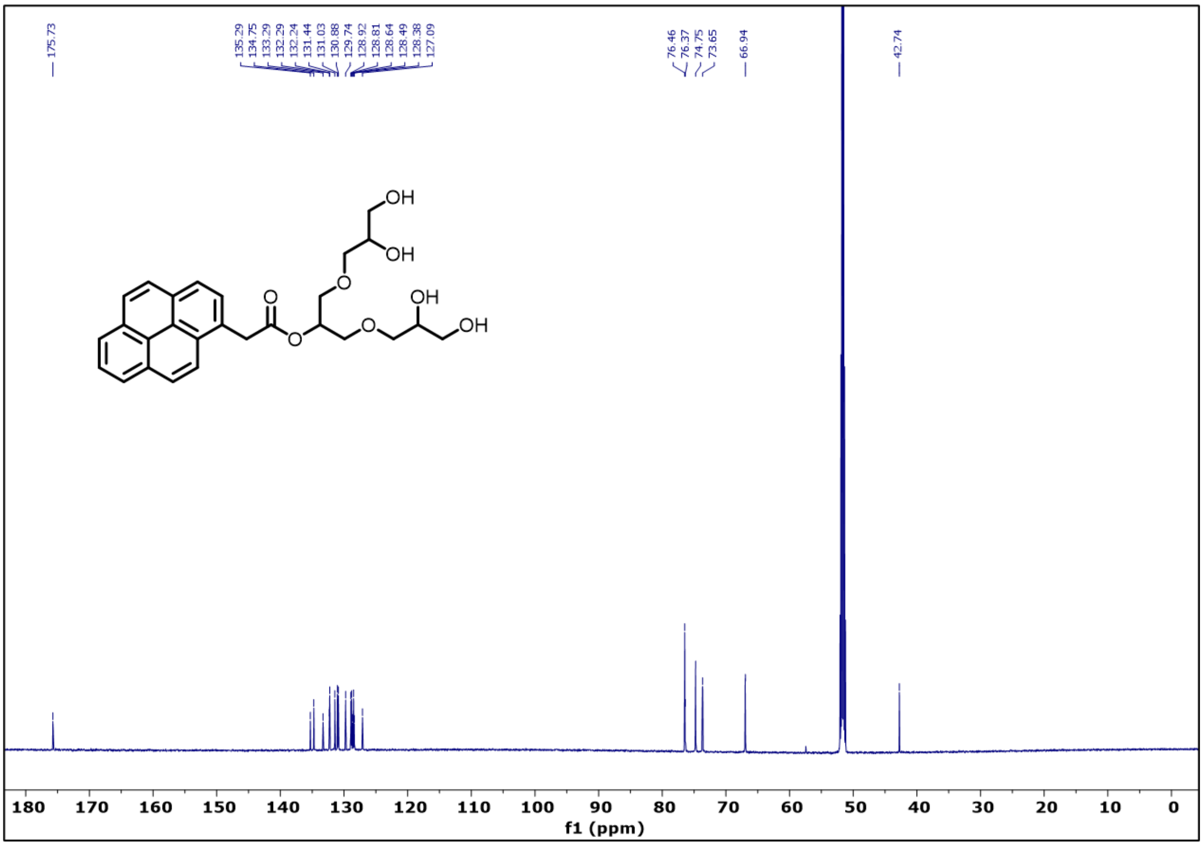

Figure S4. ^{1}H and ^{13}C NMR spectra of amphiphile Py-G1.

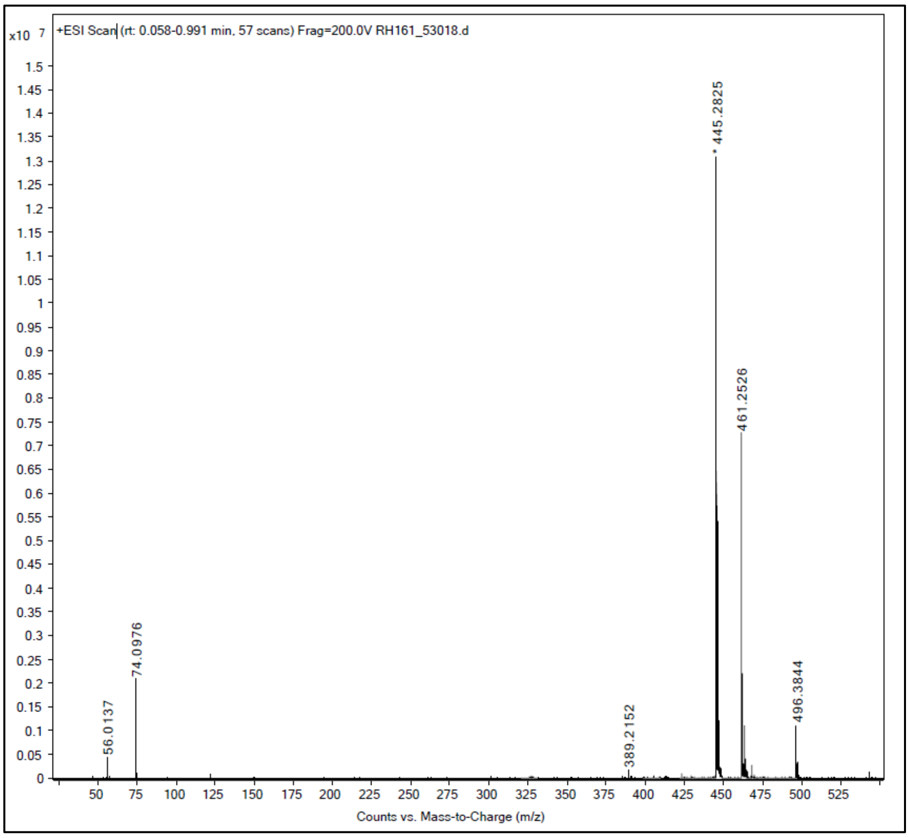

Figure S5. HRMS of amphiphile C12-G1.

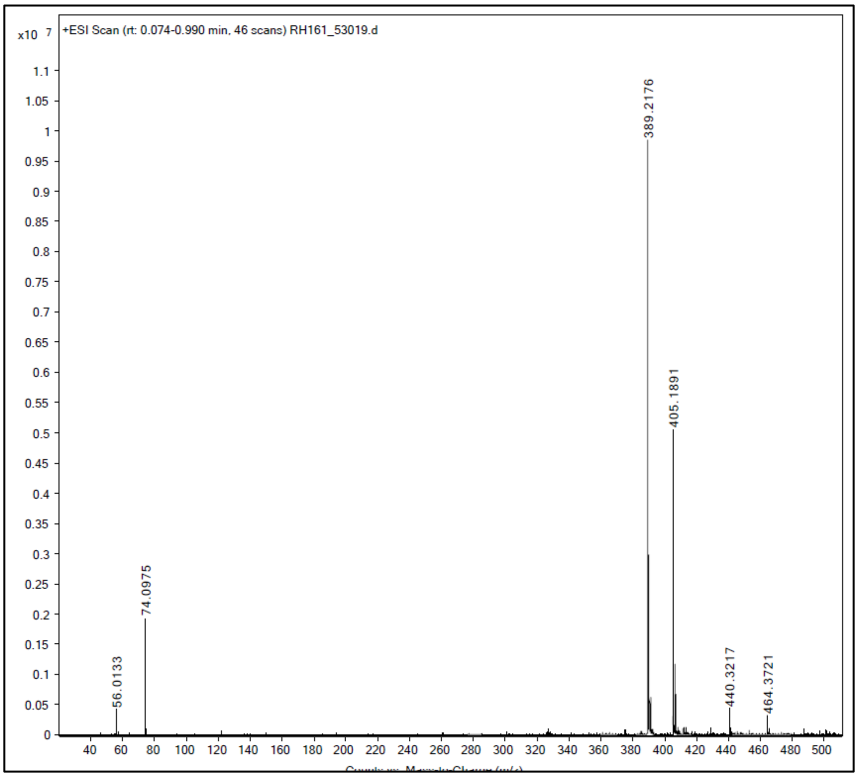

Figure S6. HRMS of amphiphile 8-G1.

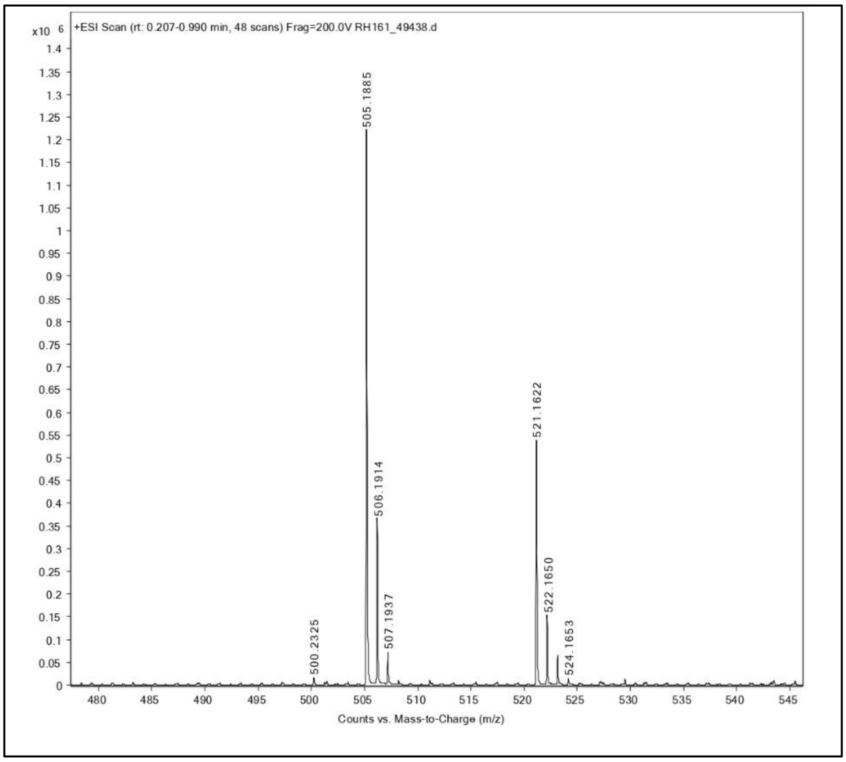

Figure S7. HRMS of amphiphile Nap-G1.

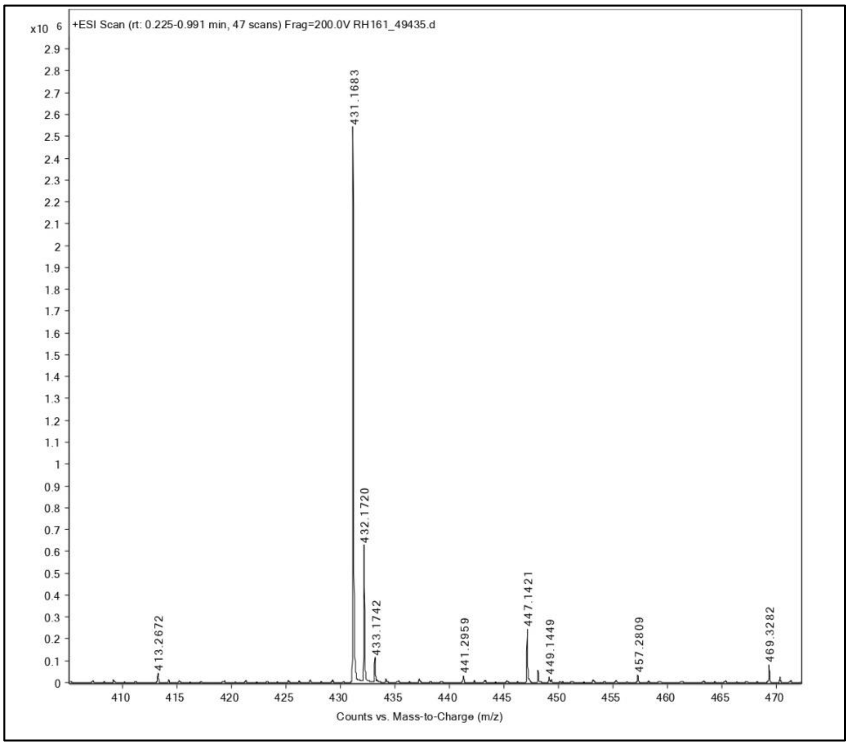

Figure S8. HRMS of amphiphile Py-G1.

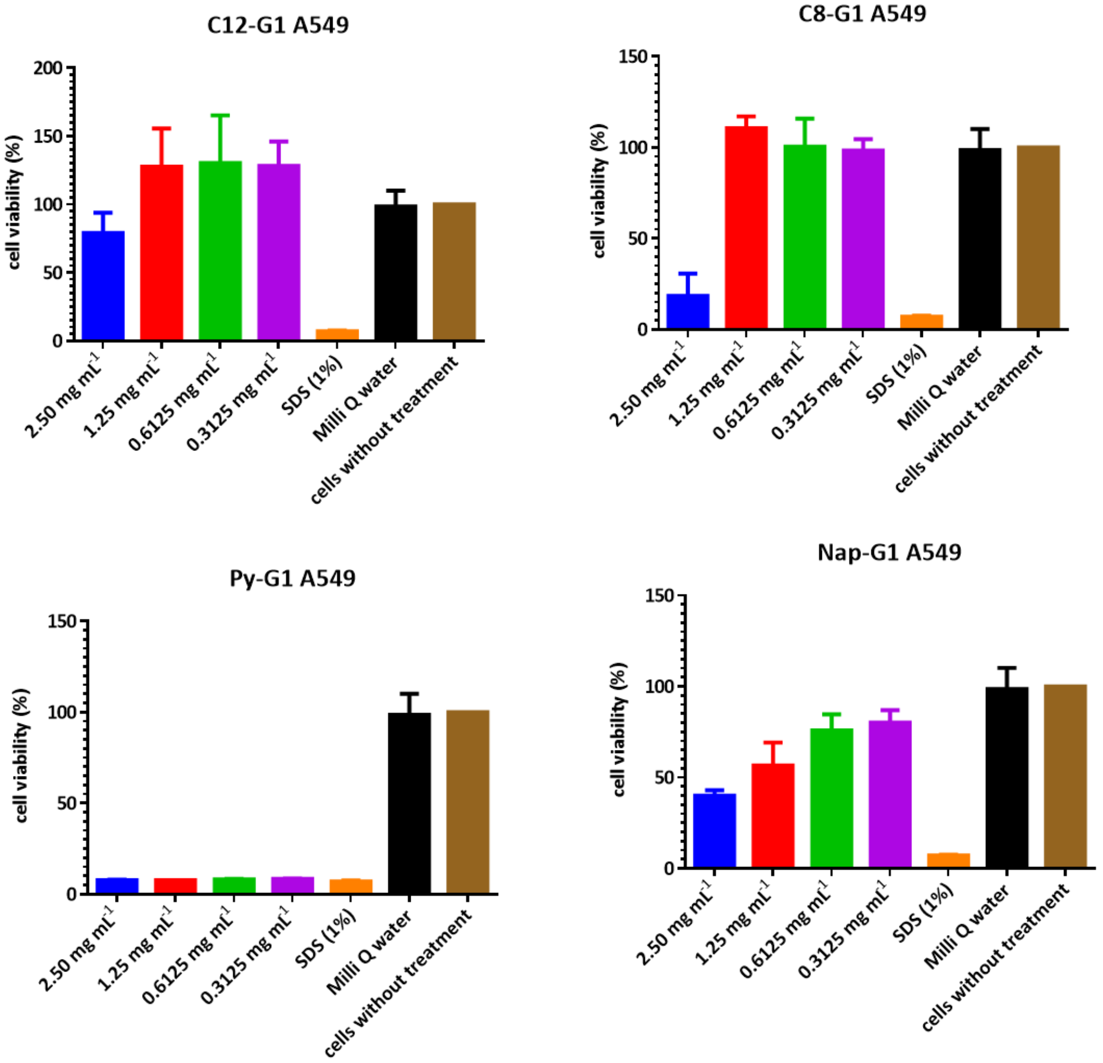

FigureS9: The correlation graph for the synthesized amphiphiles.

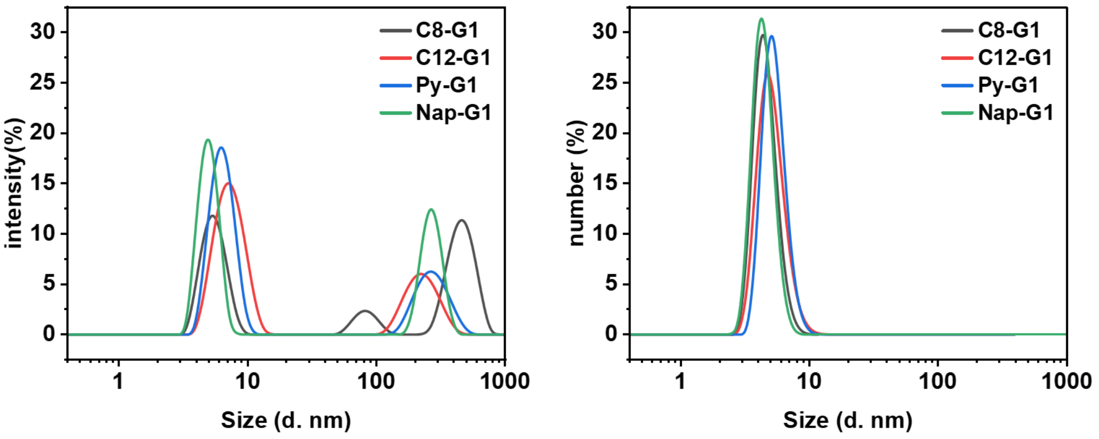

FigureS10: The correlation graph for the synthesized amphiphiles.

TableS1: The quantification of encapsulated and nonencapsulated Nile red and Nimodipine.

**Amphiphiles**	**Amount of dye/drug Used for encapsulations for 5 mg of amphiphile**		**Encapsulated amount For 5 mg of amphiphiles**		**Non-Encapsulated amount 5 mg of amphiphiles**	
	Nile Red	Nimodipine	Nile Red	Nimodipine	Nile Red	Nimodipine
C12-G1	0.2 mg	1 mg	0.0045	0.026	0.195	0.974
C8-G1	0.2 mg	1 mg	0.002	0.022	0.198	0.978
Py-G1	0.2 mg	1 mg	0.0009	0.007	0.199	0.992
Nap-G1	0.2 mg	1 mg	0.006	0.048	0.194	0.951

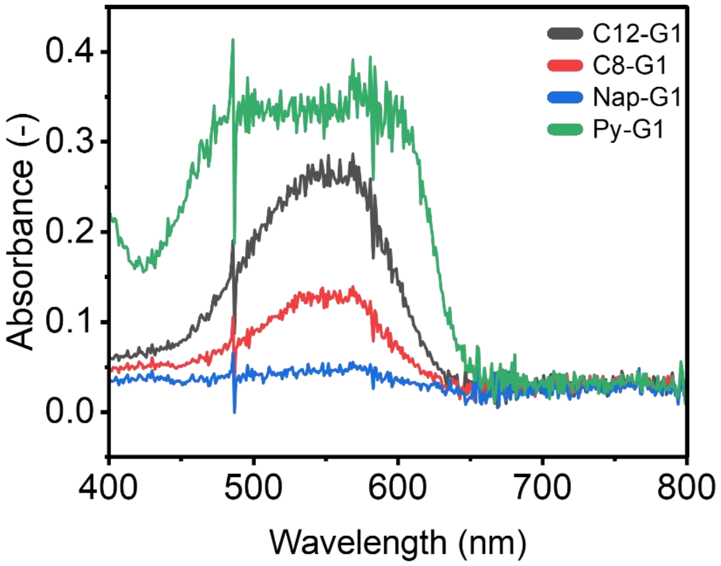

Figure S11. UV graph for Nile Red encapsulation.

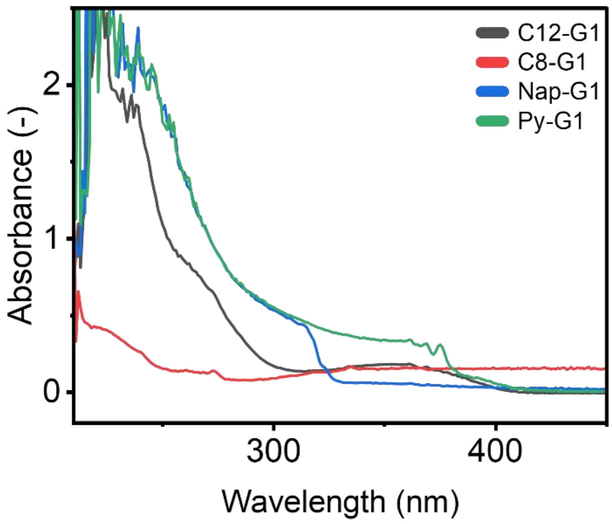

Figure S12. UV graph for Nimodipine encapsulation.
